# Gene family expansions and contractions are associated with host range in plant pathogens of the genus *Colletotrichum*

**DOI:** 10.1186/s12864-016-2917-6

**Published:** 2016-08-05

**Authors:** Riccardo Baroncelli, Daniel Buchvaldt Amby, Antonio Zapparata, Sabrina Sarrocco, Giovanni Vannacci, Gaétan Le Floch, Richard J. Harrison, Eric Holub, Serenella A. Sukno, Surapareddy Sreenivasaprasad, Michael R. Thon

**Affiliations:** 1 Laboratoire Universitaire de Biodiversité et Ecologie Microbienne (LUBEM), University of Western Brittany, Technopôle Brest-Iroise, 29280 Plouzané, France; 2 Department of Plant and Environmental Sciences and Copenhagen Plant Science Centre, University of Copenhagen, Thorvaldsensvej 40, 1871 Frb. C, Copenhagen, Denmark; 3 Department of Agriculture, Food and Environment, University of Pisa, Via del Borghetto 80, 56124 Pisa, Italy; 4 NIAB-EMR, New Road, East Malling, Kent, ME19 6BJ UK; 5 School of Life Sciences, Warwick Crop Centre, University of Warwick, Wellesbourne, Warwickshire CV35 9EF UK; 6 Instituto Hispano-Luso de Investigaciones Agrarias (CIALE), University of Salamanca, Campus de Villamayor, C/Del Duero, 12, 37185 Villamayor Salamanca, Spain; 7 Institute of Biomedical and Environmental Science and Technology (iBEST), University of Bedfordshire, University Square, Luton, Bedfordshire LU1 3JU UK

**Keywords:** CAZyme, Plant pathogen, Anthracnose, Fungal genomics, *Colletotrichum* spp.

## Abstract

**Background:**

Many species belonging to the genus *Colletotrichum* cause anthracnose disease on a wide range of plant species. In addition to their economic impact, the genus *Colletotrichum* is a useful model for the study of the evolution of host specificity, speciation and reproductive behaviors. Genome projects of *Colletotrichum* species have already opened a new era for studying the evolution of pathogenesis in fungi.

**Results:**

We sequenced and annotated the genomes of four strains in the *Colletotrichum acutatum* species complex (CAsc), a clade of broad host range pathogens within the genus. The four CAsc proteomes and secretomes along with those representing an additional 13 species (six *Colletotrichum* spp. and seven other Sordariomycetes) were classified into protein families using a variety of tools. Hierarchical clustering of gene family and functional domain assignments, and phylogenetic analyses revealed lineage specific losses of carbohydrate-active enzymes (CAZymes) and proteases encoding genes in *Colletotrichum* species that have narrow host range as well as duplications of these families in the CAsc. We also found a lineage specific expansion of necrosis and ethylene-inducing peptide 1 (Nep1)-like protein (NLPs) families within the CAsc.

**Conclusions:**

This study illustrates the plasticity of *Colletotrichum* genomes, and shows that major changes in host range are associated with relatively recent changes in gene content.

**Electronic supplementary material:**

The online version of this article (doi:10.1186/s12864-016-2917-6) contains supplementary material, which is available to authorized users.

## Background

Plant pathogenic fungi exhibit remarkable differences in the number and diversity of hosts they are able to colonize and/or infect. Based on their host range, phytopathogenic fungi can be categorised as specialists infecting a single plant or a small group of closely related plants (narrow host range), generalists associated with a wide variety of plants in diverse environments (broad host range), and transitional species capable of infecting a limited range of plants (intermediate host range). What is remarkable is the existence of plant pathogens manifesting these host range categories in the same phylogenetic lineage or different lineages within a single genus as exemplified by the globally important fungal genus *Colletotrichum* [1, 2]. Host range shifts are also intricately linked to speciation and are potentially driven by changes in lifestyle [2, 3]. Understanding the molecular determinants of the host range alternations has major implications in global food security including crop disease management, and control of pathogen introductions into new environments.

*Colletotrichum* species exhibit endophytic and/or pathogenic associations with a wide variety of herbaceous and woody plants in tropical, subtropical and temperate climates in natural and agricultural ecosystems [1, 2]. The economic impact of crop-losses caused by *Colletotrichum* pathogens has been well recognized [1, 4]. Recent multi-locus phylogenetic studies of the genus *Colletotrichum* led to the identification of at least 10 major clades such as acutatum, gloeosporioides and boninense including at least 28, 22 and 17 species, respectively [2]. *Colletotrichum* species identified within and among these major clades or lineages exhibit remarkable differences in their host range. Within the *C. acutatum* species complex (CAsc), species such as *C. nymphaeae*, *C. simmondsii* and *C. fioriniae* display broad host range, *C. salicis* an intermediate range of woody hosts [5], and *C. lupini* a narrow host range for lupins [6, 7]. A similar pattern can be found among species belonging to the *C. gloeosporioides* and *C. boninense* species complexes. Conversely, the *C. orbiculare* complex includes species with a narrow host range [8–11]. The trajectory of evolution of specialists and generalists in *Colletotrichum* pathogens, and how this change is mirrored in the genomic architecture of various species remain to be addressed.

Since the first genome sequences of phytopathogenic fungi became available, researchers have been analyzing gene content to find associations that may explain the differences in fungal lifestyles [12] and varying patterns are beginning to emerge. Some studies have suggested that differences in gene family size are more strongly associated with phylogenetic relatedness than lifestyle [13]. In contrast, other studies have found a larger number of secreted enzymes in pathogens compared to non-pathogens, and also in nectrotrophic and hemibiotrophic fungi compared to biotrophs [14–18]. These studies suggest that specific patterns of gene content may be associated with the adaptation of diverse fungal lifestyles.

In this manuscript, we report the genome sequences of four *Colletotrichum* species representing the diversity within the CAsc, and the comparative analysis with the genome sequences of species representing narrow, intermediate and broad host ranges from other major clades/lineages. We studied differences in gene content between the species by focusing our analyses on two classes of proteins known to have roles in plant - pathogen interactions: secreted proteins and enzymes responsible for secondary metabolite biosynthesis. Comparative genomics revealed contractions of gene families encoding carbohydrate active (CAZymes) and proteolytic enzymes specifically within host-specific species as well as lineage specific expansions with the CAsc. We also found an expansion of necrosis and ethylene-inducing peptide 1 (Nep1)-like proteins (NLPs) and a contraction of Lineage Specific Effector protein Candidates (LSECs) within CAsc. Based on these patterns, we hypothesize that in broad host range *Colletotrichum* species particularly within CAsc, LSECs have reduced roles in plant interactions and that they may instead rely on CAZymes, proteases and NLPs for host colonization. This study demonstrates the utility of higher resolution sampling for comparative genomic studies in the filamentous fungal pathogens.

## Methods

### Evolutionary relationships in *Colletotrichum* spp.

A phylogeny of the genus *Colletotrichum* was constructed based on publicly available DNA sequences of four nuclear loci: part of the ribosomal RNA gene cluster (rRNA) (ITS1-5.8S-ITS2), beta tubulin (βTUB) partial sequence, actin (ACT) partial sequence and glyceraldehyde-3-phosphate dehydrogenase (GAPDH) partial sequence. A complete list of the sequences used is reported in Additional file 1: Table S1. All the sequences were aligned using MAFFT 7 [19] and the multiple sequence alignments were exported to MEGA 6.06 [20] where best-fit substitution models were calculated for each separate sequence dataset. The concatenated alignment (ITS, TUB2, ACT and GAPDH) was performed with Geneious 8.1.4. A Markov Chain Monte Carlo (MCMC) algorithm was used to generate phylogenetic trees with Bayesian probabilities using MrBayes 3.2.1 [21]. Models of nucleotide substitution for each gene determined by MEGA 6.06 [20] were included for each locus. The analyses of four MCMC chains were run from random trees for 5000000 numbers of generations and sampled every 100 generations.

### Fungal strains

*C. simmondsii* - CBS 122122 also known as BRIP 28519; HKUCC 10928; ICMP 17298; KACC 43258). This strain was collected during May of 1987 by L.M. Coates in Queensland, Australia from infected fruit tissues of papaya [sn: *Carica papaya*]. The strain has been nominated as the holotype of the species [5].

*C. fioriniae* [22] - IMI 504882 also known as PJ7 was isolated by Peter R. Johnston from infected strawberry [sn: *Fragaria* x *ananassa*] fruit in the Auckland area, New Zealand in 1988 [23]. The strain has been used as a reference strain for phylogenetic analyses of the CAsc and for mating tests and pathogenicity assays [24]. Heterothallic mating capability of this strain has been demonstrated in laboratory experiments [24].

*C. nymphaeae* – IMI 504889 also known as SA01 collected by T. Sundelin in June 2000 in Denmark in a production field on the island of Falster from infected strawberry plants of cv. Kimberly [25]. The strain has been used as a model to study *Colletotrichum*/strawberry interaction with an integrated ‘omics approach. *C. nymphaeae* is the most wide distributed specie of CAsc and is one of the most important pathogen on different crops such strawberry and olive [26, 27]. A sexual state has not been reported.

*C. salicis* – CBS 607.94 isolated in 1994 by H.A. van der Aa from infected leaf tissue of Salix sp. in the Salix Forest near Blocq van Kuffeler, Netherlands. Originally epitype of *Sphaeria salicis*, now designated, culture ex-epitype for *C. salicis* [5]. Synonymous of *Glomerella miyabeana* isolates belonging to this species are homothallic [23, 28].

### DNA extraction

Genomic DNA was extracted based on a modified cetyltrimethylammonium-bromide (CTAB) procedure [29]. The mycelium (250 mg) was ground under liquid nitrogen using a presterilized chilled mortar and pestle. The resultant powder was mixed with 15 ml of a preheated solution (60 °C) containing 10 % CTAB, 2 M Tris-CI (pH 8.0), 0,5 M EDTA, 1.4 M NaCI and 0,5 % 2-mercaptoethanol. After incubation for 30 min at 60 °C, proteins were removed twice with 15 ml volume of chloroformisoamyl alcohol 24:1 (*v/v*). The aqueous phase was transferred to a clean tube, and the nucleic acids were precipitated with 0.6 volume of cold 2-propanol. After 2-hour incubation at room temperature, the samples were centrifuged for 2 min at 460 g. The pellet was washed twice with 66 % (*v/v*) EtOH and 34 % of 0.1 M NaCl. Tubes were centrifuged at 1500 g for 10 min, Washing buffer (supernatant) was removed and pellet were air dried in the fume hood (approximately 1 h). The pellet was resuspended in one ml of AB, left for few minutes, centrifuged for 5 min and supernatant (DNA) saved and pellet discarded.

### Genome sequencing and assembly

Fifty microliters samples of genomic DNA (20 μg/μl) in AB buffer quantified with PicoGreen on a Qubit 2.0 Fluorometer were used for library preparation and sequencing. The samples were sequenced on Illumina GAII and MiSeq instruments using different library preparation and sequencing kits (Table 1).

**Table 1:** Summary of sequence data sets generated for CAsc genome sequencing

	GAII		MiSEQ		
	70 bp	50 bp	250 bp	Tot N bp	Coverage
*C. fioriniae*	6.84E + 08	1.75E + 09	-	2.44E + 09	43.03
IMI 504882					
*C. simmondsii*	1.97E + 09	-	-	1.97E + 09	33.33
CBS 122122					
*C. salicis*	2.89E + 08	6.83E + 08	1.05E + 09	2.02E + 09	36.40
CBS 607.94					
*C. nymphaeae*	-	-	2.35E + 09	2.35E + 09	42.08
IMI 504889					

For the samples sequenced on the Illumina GAII, genomic libraries with an average insert size of 260 bp were constructed using TruSeqTM RNA and DNA sample preparation kits (Illumina Inc.). Library preparation and sequencing of the 70 base, paired end libraries was carried out at the School of Life Sciences of the University of Warwick. The 50 base, paired end libraries were prepared and sequenced at the Wellcome Trust Sanger Institute. The 250 base paired end MiSeq libraries were prepared using the Nextera DNA Sample Prep Kit and sequenced at the NIAB-EMR (East Malling, Kent, UK).

The genome assemblies were performed using Velvet 1.2.10 [30] after optimizing k-mer values within the range of 21 to 69. The k-mer found to give the best results based on N50 statistics was used to produce the final assemblies (39 for *C. salicis*, 31 for *C. fioriniae* and *C. simmondsii* and 65 for *C. nymphaeae*). Only scaffolds longer than 200 bp and with k-mer coverage higher than 10X were retained in the final genomic assembly. The overall genome coverage (Table 1) was estimated using the peak on the k-mer frequency distribution curve reported by Jellyfish 2.1.3 [31]. The contigs corresponding to the mitochondrial genome (mtDNA) and the ribosomal RNA encoding gene cluster were identified by BLASTN searches using the *C. graminicola* mitochondrial genome as the query sequence. Each mitochondrial genome and rRNA cluster were assembled into one scaffold and removed from further analyses. The completeness of the assembly was assessed using BUSCO v1.2 [32].

### Gene structure annotation

The MAKER2 annotation pipeline [33] was used to annotate the CAsc genome. Two different sets of assembled sequences from transcriptomic samples were used as EST evidence. The first library belonging to *C. fioriniae* conidial germination stage and available in GenBank (EST: LIBEST_024551) the second library belonging to *C. nymphaeae* SA-01 during plant interaction (kindly provided by Birgit Jensen, Department of Plant and Environmental Sciences, University of Copenhagen, Denmark).

Protein evidence included fungal proteins downloaded from UniProtKB/SwissProt (release 2013_12) (Uniprot [34]). Three different *ab initio* gene annotation programs were trained for use with MAKER2. GeneMark-ES [35] was self-trained for each of the four genomes. SNAP [36] was trained following the protocol included with the documentation using transcriptome sequences. AUGUSTUS [37] was used with gene model belonging to closely related organism *Fusarium graminearum.*

Putative functions were assigned to the annotations by using BLASTP [38] to identify homologs in a database constructed of proteins from the UniProt database (release 2013_12) from *Neurospora crassa*, *Magnaporthe oryzae*, *Fusarium oxysporum*, *Fusarium graminearum*, *Verticillium alfalfae*, *Verticillium dahliae*, *C. graminicola*, and *C. higginsianum*.

### Comparative genomics

The genomes of twelve additional fungal species belonging to the Sordariomycetes, including all publicly available *Colletotrichum* spp. were also included the analyses (Table 2).

**Table 2:** List of species used in this study. In bold are highlighted the four *Colletotrichum acutatum* species presented in this work

Strain	Organisms	Abbr.	Host	Origin	Accession N°	Project N°	Ref.
**IMI 504889**	***C. nymphaeae***	***CNYM***	***Fragaria x ananassa***	**Denmark**	**JEMN00000000.1**	**PRJNA237763**	-
**CBS 122122**	***C. simmondsii***	***CSIM***	***Carica papaya***	**Australia**	**JFBX00000000.1**	**PRJNA239224**	-
**IMI 504882**	***C. fioriniae***	***CFIO***	***Fragaria x ananassa***	**New Zealand**	**JARH00000000.1**	**PRJNA233987**	[22]
**CBS 607.94**	***C. salicis***	***CSAL***	***Salix sp.***	**Netherlands**	**JFFI00000000.1**	**PRJNA238477**	-
M1.001	*C. graminicola*	*CGRA*	*Zea mays*	USA	ACOD00000000.1	PRJNA37879	[17]
TX430BB	*C. sublineola*	*CSUB*	*Sorghum bicolor*	USA	JMSE00000000.1	PRJNA246670	[100]
IMI 349063	*C. higginsianum*	*CHIG*	*Brassica rapa*	Trinidad & Tobago	CACQ00000000.2	PRJNA47061	[17]
Nara gc5	*C. fruticola*	*CFRU*	*Fragaria x ananassa*	Japan	ANPB00000000.1	PRJNA171218	[65]
Cg-14	*C. gloeosporioides*	*CGLO*	*Persea americana*	Israel	AMYD00000000.1	PRJNA176412	[101]
MAFF 240422	*C. orbiculare*	*CORB*	*Cucumis sativus*	Japan	AMCV00000000.1	PRJNA171217	[65]
VaMs.102	*V. alfalfae*	*VALF*	*Medicago sativa*	USA	ABPE00000000.1	PRJNA51263	[102]
VdLs.17	*V. dahliae*	*VDAH*	*Lactuca sativa*	USA	ABJE00000000.1	PRJNA28529	[102]
PH1	*F. graminearum*	*FGRA*	*Triticum sp.*	USA	AACM00000000.2	PRJNA13839	[103]
FOL 4287	*F. oxysporum*	*FOXY*	*Solanum lycopersicum*	Spain	AAXH00000000.1	PRJNA18813	[103]
20.1	*Cl. purpurea*	*CPUR*	*Secale cereale*	Germany	CAGA00000000.1	PRJEA76493	[47]
70-15	*M. oryzae*	*MORY*	*Oryza sativa*	French Guiana	AACU00000000.3	PRJNA16061	[12]
or74a	*N. crassa*	*NCRA*	no pathogen	USA	AABX00000000.3	PRJNA13841	[104]

**Fig. 1:**
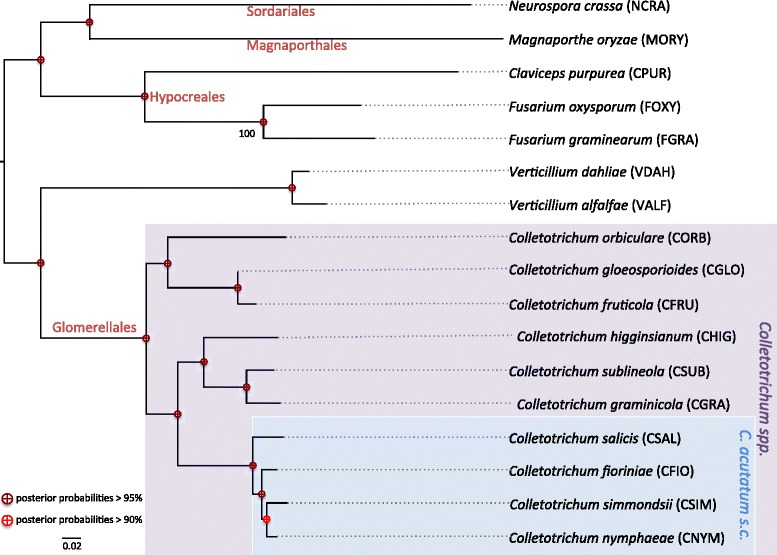
Evolutionary relationships among ten *Colletotrichum* species and seven additional species used for comparative analyses. The tree was constructed using Bayesian MCMC analysis constructed from the alignment based on the concatenated alignment of the five most phylogenetically informative single copy gene families

The proteomes were clustered OrthoFinder v0.4 [39] and the clusters were analysed with Mirlo (https://github.com/mthon/mirlo) to identify the five most phylogenetically informative single copy gene families. The five families were aligned with MAFFT 7 [19] and then concatenated. A substitution model and its parameter values were selected using ProtTest 3.4 [40]. A phylogenetic tree was reconstructed using Bayesian MCMC analysis constructed from the alignment based on the concatenated alignment under the WAG + I evolutionary model and the gamma distribution calculated using four rate categories and homogeneous rates across the tree. The posterior probabilities threshold was selected as over 75 % (Fig. 1).

### Functional annotation

#### Prediction and analyses of secretomes

Proteins that are transported out of the cell and into the extracellular space were identified with WoLF-PSORT [41] as described previously [17]. The final set of putative secreted proteins were scanned with InterProScan [42] using RunIprScan 1.1.0 (http://michaelrthon.com/runiprscan/) to identify protein domain signatures and relative changes in selected genomes.

#### Secreted carbohydrate active enzymes (CAZymes)

We used the Hmmscan program in the HMMER 3.0 package [43] to search each of fungal predicted proteomes with the family-specific HMM profiles of CAZymes downloaded from dbCAN database 2.0 [44] with cut-off E-value of 1E-3 as queries. The primary results were processed and checked manually: overlapping matches removed and cut-off E-value adjusted based on comparison with the manually curated *C. graminicola* CAZome. For each CAZy class, the number of enzyme modules and the families they belong to are reported.

#### Secreted proteases

Predicted proteins were classified as proteases by querying the MEROPS database 10.0 [45] using a BLASTp cut-off E-value of 1E-5. Sequences with similarity to protease domains but with mutated active sites and incomplete protease domains were further excluded as proteases.

#### Necrosis and ethylene-inducing peptide 1 (Nep1)-like proteins (NLPs)

The CaNLP genes were identified in the *Colletotrichum* spp. genomes by searching a six-frame translation on the conserved GHRHDWE motif [46]. RunIprScan was also used to scan predicted secretomes to identify genes encoding NLPs (IPR008701/PF05630).

### Identification of lineage specific effector protein candidates (LSECs)

We defined LSECs as proteins with no (detectable) homology to any other protein. We also exclude proteins with conserved domains, as a conserved domain may imply shared ancestry. We identified LSECs by performing BLAST searches to the GenBank nr BLAST database using an e-value threshold of 1e-5. Proteins with homology to proteins from other members of the same genus but not to other genera were termed ‘genus-specific.’ Those that had no homology to any other protein either within or outside of the same genus were termed ‘species-specific.’

### Secondary metabolites related genes and clusters

BLASTp and RunIprScan were used to manually identify genes encoding enzymes that are signatures of backbone genes and secondary metabolite (SM) gene in the Ascomycota: nonribosomal peptide synthetases (NRPS; IPR010071, IPR006163, IPR001242), polyketide synthases (PKS; IPR013968), DMATS-family aromatic prenyltransferases (IPR017795, Pfam PF11991), and terpene synthases/cyclases (IPR008949) [47]. AntiSMASH version 1.2.2 [48] was downloaded and run locally on all genomes analysed in order to identify secondary metabolite gene clusters. The predicted clusters were visually evaluated for conservation of synteny by examining whether similar block of genes related to SM biosynthesis are orthologs in the other species. In cases where a gene in the species in which the cluster was identified no longer had orthologs in the other species, we inferred a break in synteny.

## Results

### Phylogenetic analyses

To better understand the evolutionary relationships among species within the genus *Colletotrichum*, we performed a phylogenetic analysis using four loci obtained from 133 isolates (Additional file 2: Figure S1). It is now well established that the genus *Colletotrichum* consists of ten major monophyletic clades that are referred to as species complexes [2, 49], all of which are present and well supported by our phylogenetic analysis. Although species complexes have no taxonomic value, they do provide a useful summary of knowledge to link evolutionarily related species within a genus.

The number of clades may increase as several other groups have been identified in the analyses and existing clade with very few species have already been described. For example, the truncatum clade includes one major species (*C. truncatum*) and two poorly-known species (*C. curcumae* and *C. jasminigenum*); the second species has been removed from the analyses because the only existing isolate seems to be originated by hybridization event between one isolate of *C. truncatum* and one belonging to the CGsc (based on data available on GenBank).

The CAsc and CGsc have the highest number of species among the species complexes of *Colletotrichum* and they appear to be evolutionary very distant despite the similarities in morphology and host range. Both of these species complexes contain broad host range species but representative genome sequences are only available for the CGsc*.* Therefore, we selected four isolates from the CAsc for whole genome shotgun sequencing. The four isolates chosen represent the genetic diversity of the CAsc and are also commonly used in research laboratories as references for evolutionary analyses, phylogenetics and pathogenicity assays.

### Genome sequencing, assembly and gene prediction

Genome sequencing was performed with the Illumina Genome Analyzer IIx or MiSeq sequencers and assembled into scaffolds with Velvet 1.2.10 [30]. Contigs representing the mitochondrial genomes were identified with BLAST and removed from the assemblies. The nuclear genome assembly sizes ranged from 48 to 50 Mb (Table 3), which are comparable to the other sequenced *Colletotrichum* genomes. Benchmarking Universal Single-Copy Orthologs (BUSCO v. 1.2; [32]) was used to provide an estimate of assembly completeness. According to this analysis, the assemblies for the CAsc genomes cover from 99.79 to 99.93 % of the total gene space, which is comparable to other sequenced fungal genomes (Table 3). Gene models were annotated with MAKER2. Secreted proteins were identified using WoLF-PSORT [41] (Table 3).

**Table 3:** Assembly and gene prediction information of *Colletotrichum* spp. genomes and of other fungi chosen as out-group

Abbr.	N° Scaffolds	Assembly Size	max contig	N50	N90	BUSCO complete	BUSCO partial	Proteome size	Secretome size	% of secreted proteins
*CNYM*	1884	50	494648	91051	19816	98.54 %	99.79 %	14404	2244	15.58 %
*CSIM*	929	50	802711	292136	87006	99.17 %	99.93 %	13884	2211	15.92 %
*CFIO*	1096	49	596408	137254	38253	99.10 %	99.93 %	13759	2200	15.99 %
*CSAL*	2776	48	217493	46166	10883	98.75 %	99.79 %	13783	2126	15.42 %
*CGRA*	654	52	1824042	579194	37593	99.17 %	99.86 %	12006	1650	13.74 %
*CSUB*	1625	47	423147	70717	13454	99.03 %	99.86 %	12699	1820	14.33 %
*CHIG*	10235	49	49362	6147	2360	87.48 %	98.40 %	16172	2136	13.21 %
*CFRU*	1241	56	493961	112809	28004	94.92 %	99.37 %	15463	2356	15.24 %
*CGLO*	4537	53	128447	25337	7246	99.30 %	97.43 %	15736	2376	15.10 %
*CORB*	526	91	2513218	449288	92779	99.03 %	99.86 %	13479	2149	15.94 %
*VALF*	26	33	4782674	2315232	1129871	84.49 %	96.31 %	10220	1310	12.82 %
*VDAH*	52	34	2667998	1273651	499255	96.52 %	99.10 %	10535	1383	13.13 %
*FGRA*	31	36	8931406	5350016	2732284	98.75 %	99.79 %	13321	1542	11.58 %
*FOXY*	114	61	4358238	1976106	500264	97.91 %	99.51 %	17701	1935	10.93 %
*CPUR*	191	32	977986	433221	110809	98.96 %	99.44 %	8823	871	9.87 %
*MORY*	30	42	4429722	2890137	851181	98.96 %	99.65 %	11054	1832	16.57 %
*NCRA*	20	41	9798893	6000761	4218384	98.96 %	99.86 %	9907	1040	10.50 %

### Genome-wide analysis of gene content

We scanned the annotated proteins of the CAsc genomes with RunIprScan to identify conserved functional domains and families described in the Interpro database. We compared their InterPro (IPR) domain content to those of other *Colletotrichum* species and a representative set of closely related fungi.

**Fig. 2:**
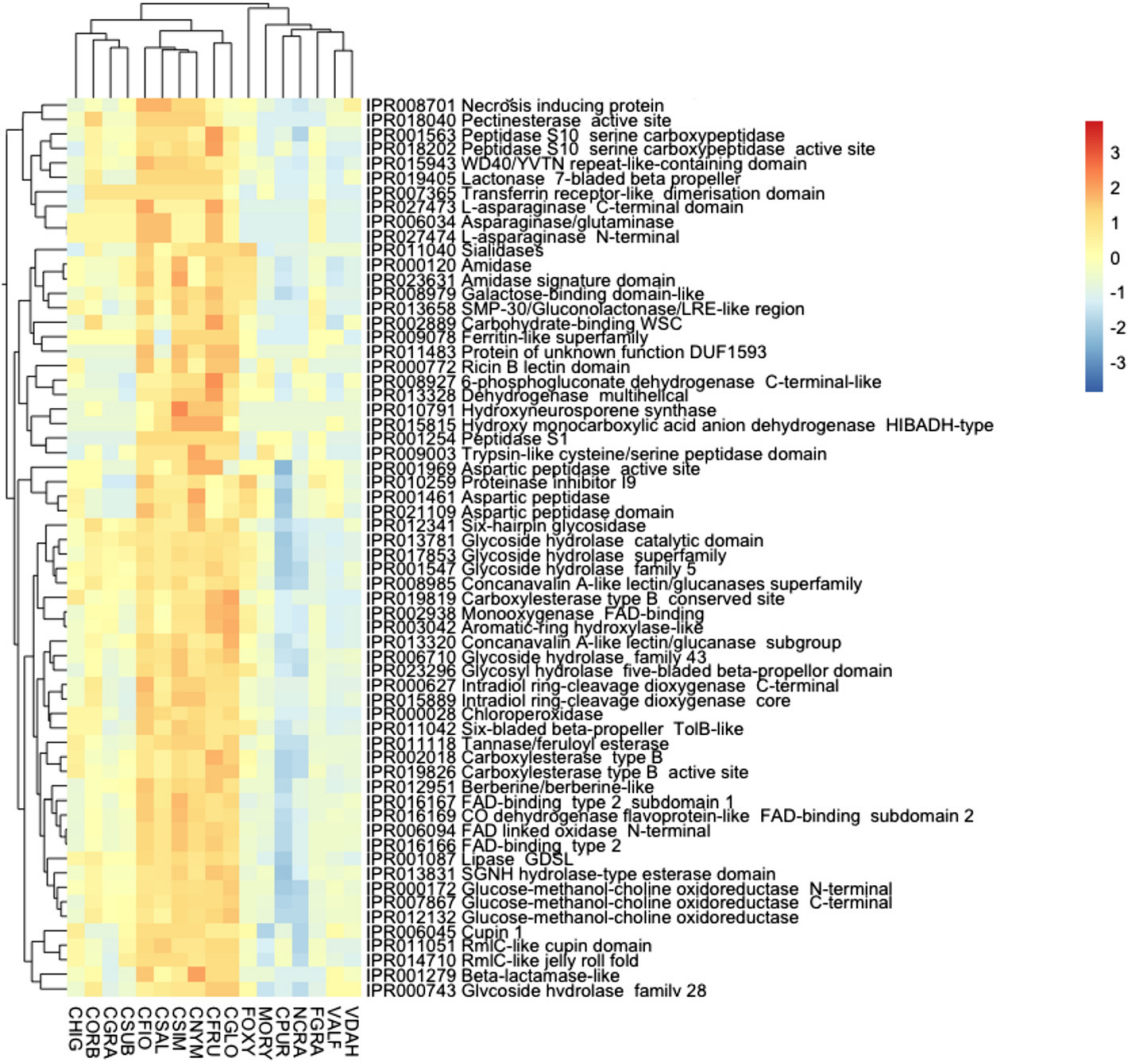
Hierarchical clustering of IPR terms expanded in *Colletotrichum acutatum* and *C. gloeosporioides* species complexes compared to other *Colletotrichum* species and other model fungal genomes. Number of genes characterized by each IPR has been normalized using MeV 4.8.1. Hierarchical clustering of genes and species was performed and visualized using the package “pheatmap” 1.0.8 within R Overrepresented (*orange* to *red*) and underrepresented functional domains (*blue*) are depicted as fold changes relative to the IPR term mean

We performed hierarchical clustering of the IPR terms of each species (Additional file 3: Table S2) to identify genome-wide patterns of functional domain content. Hierarchical clustering of the CAsc isolates with 13 other fungi belonging to *Colletotrichum* and other spp. revealed that the CAsc isolates clustered with the two CGsc isolates. This result was unexpected since it has been previously shown that gene family content in fungi is more closely associated with phylogeny than lifestyle [13] and these two species complexes are not closely related phylogenetically (Additional file 2: Figure S1). Hierarchical clustering was also performed on the IPR domain content of the predicted secretomes showing the same pattern and manual inspection revealed a cluster of overrepresented IPR terms in the CAsc and CGsc lineages, containing 62 IPR terms (Fig. 2). Of these IPR terms, the majority have roles in carbohydrate metabolism and protein degradation and one is characterized by a “necrosis inducing protein” conserved domain. We further studied differences in gene content within these three gene classes by performing manual annotation of the gene families followed by additional hierarchical clustering and phylogenetic analyses.

### Secreted carbohydrate active enzymes

CAZymes (Carbohydrate Active enZymes) are proteins involved in the degradation, rearrangement, or synthesis of glycosidic bonds [50]. So far only a few cell wall-degrading enzymes (CWDEs) have been reported as having an important role in pathogenicity [51], probably due to the genetic redundancy of these genes. However, CAZymes are essential to establish a relationship with the host and in degradation of plant biomass in order to gain nutrition [14].

**Fig. 3:**
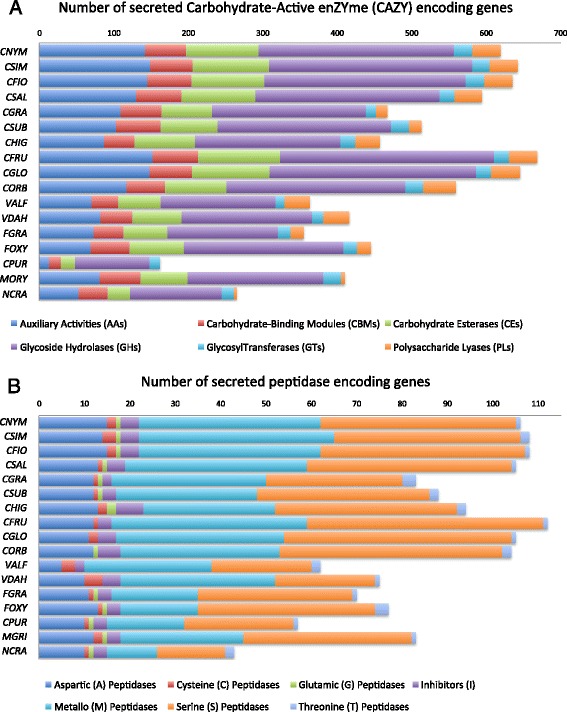
**a** Distribution of secreted enzymes belonging to each CAZy (Carbohydrate Active enZymes) class identified in the genomes used in this study. The legend on the bottom reports the designation of enzyme classes: Glycoside Hydrolases (GH), Glycosyl Transferases (GT), Polysaccharide Lyases (PL), Carbohydrate Esterases (CE), Auxiliary Activities (AA). The legend on the bottom reports the designation of enzyme classes: Glycoside Hydrolases (GH), Glycosyl Transferases (GT), Polysaccharide Lyases (PL), Carbohydrate Esterases (CE), Auxiliary Activities (AA). **b** Extracellular secreted protease homologs classified according to the MEROPS database 10.0 [45] in *Colletotrichum* and other fungal genomes used in this study

The heatmap shown in Fig. 2 revealed that the CAsc and CGsc genomes have higher copy numbers of several CAZyme families than the other *Colletotrichum* spp., suggesting that lineage specific expansions of these families occurred during the evolution of these species. To study the evolution of the CAZymes in more detail, we first reannotated the CAZY families using the HMMER 3.0 package [43] to compare the proteomes to the DBcan database 2.0 [44] of CAZyme signature domains and then curated the results manually, performing multiple sequence alignments where necessary to confirm family membership of each protein (Additional file 4: Table S3). Among the species included in this study, the four members of the CAsc and the two members of the CGsc have the largest repertoires of CAZymes (Fig. 3a). We constructed a heatmap of the manually curated CAZy families, which also showed higher copy numbers of these families in CAsc and CGsc species (Additional file 5: Figure S2). Based on this new heatmap, we selected the six families showing the largest number of genes in CAsc and CGsc for phylogenetic analysis (Additional file 5: Figures S8–S13). Each gene tree can be divided into clades that reflect the phylogenetic relationships of the species (red for CAsc and blue for other *Colletotrichum* spp., Additional file 5: Figures S8–S13), indicating that in many cases, the gene duplications leading to the CAZyme content present in these fungi preceded the speciation events. Many of the clades that contain CAsc gene copies lack one or more other *Colletotrichum* species, indicating gene loss in these species.

All of the fungal genomes studied here encode similar numbers of glycosyltransferases (GTs), which are genes that are involved in basal activities of the fungal cell. These results are consistent with those of O’Connell et al. [17]. The families with the highest copy numbers in members of CAsc and CGsc compared to the others are genes encoding carbohydrate esterases (CEs) that catalyze the de-O or de-N-acylation of substituted saccharides, genes encoding enzymes that hydrolyse the glycosidic bond between two or more carbohydrates or between a carbohydrate and a non-carbohydrate moiety (GHs). The large reserve of sugar-cleaving enzymes is further extended by polysaccharide lyases (PLs) enzymes encoded by the *Colletotrichum* species belonging to the CAsc and CGsc*.* PLs are more overrepresented in those pathogens capable of infecting dicotyledonous such as members of CAsc and CGsc as well as *C. higginsiaum* and *C. orbiculare* compared to the monocotyledonous pathogens *C. graminicola* and *C. sublineola*.

Among the most expanded gene families in CAsc and CGsc are those involved in the degradation of pectin such as GH53, GH78, GH28, GH105, CE8, CE12, PL3 and PL1 and those involved in degradation of xyloglucan and cellulose such as GH1, GH12 and GH7 (Additional file 5: Figure S2). Families encoding xylan and pectin degrading enzymes such as GH39 and GH43 are also exhibiting higher copy numbers. This last family shows on average twice the number of genes in CAsc and CGsc compared to other organisms analyzed. A different distribution is found in the carbohydrate-binding modules family 50 (CBM50 or LysM). The number of LysM motif containing proteins varies considerably in closely related species. The number of LysM encoding genes is between 7 and 13 in the CAsc and 8 and 16 in the two genomes of CGsc.

Auxiliary Activities (AA) is another class of genes not belonging to carbohydrate-active enzymes but instead linked to biomass degradation and microbe-plant interaction (e.g.*,* involved in lignin breakdown). All *Colletotrichum* species analyzed encode a large diversity of AA genes. Also in this case species belonging to the CAsc and CGsc encode the highest number of genes belonging to this class. The AA class is a widespread group of catalytic modules involved in plant cell wall degradation and a former classification dedicated to fungal ligninolytic enzymes [52]. Particularly overrepresented are the AA1, AA7 and AA3 families (Additional file 5: Figure S2). The categorized AA1 enzymes are multicopper oxidases that use diphenols and associated constituents as donors with oxygen as the acceptor. AA3 is the glucose-methanol-choline (GMC) oxidoreductases family and are flavoproteins containing a flavin-adenine dinucleotide (FAD)-binding domain, proteins belonging to this sub-family act as cellobiose dehydrogenases, aryl alcohol oxidase, glucose 1-oxidase, alcohol oxidase and pyranose 2-oxidase. AA7 are glucooligosaccharide oxidases that oxidize the reducing end glycosyl residues of oligosaccharides linked by alpha- or beta-1,4 bonds and glucose. Identified AA7 enzymes are potentially implicated in the biotransformation or detoxification of lignocellulosic compounds [52]. The large number of AA genes encoded by species belonging to the CAsc and CGsc reflect the high ecological diversity of those organisms compared to the other species, in fact CAsc and CGsc have an impressive wide range of hosts that included trees.

### Secreted proteases

Several families of proteases were also identified as having higher copy number in the genome wide heat map analysis of Fig. 2. Therefore, to further investigate the differences in protease content between species, we curated protease gene families by performing BLASTP searches to the MEROPS database (Additional file 6: Table S4). In the CAsc genomes, approx. 28 % of putative secreted proteins were assigned to protease encoding gene families (Fig. 3b). The genomes of CAsc, CGsc and C. *orbiculare* contain the highest number of secreted peptidases compared to the other *Colletotrichum* species and other spp. (Additional file 5: Figure S3).

Among the largest protease gene families in CAsc are those showing similarities with aspartic, metallo and serine peptidase families A01, M35 and S10 (Additional file 5: Figure S3) which are characterized (along with C13, G01, and S09) by the ability to efficiently digest proteins in inhospitable environments such as the extracellular matrix [53]. Family M43 cytophagalysin zinc-dependent metalloprotease was also among the most overrepresented; however, very little information is available concerning a protective role of these genes in plant pathogens. We constructed phylogenetic trees for these families (Additional file 5: Figures S4–S7) and, as is the case with the CAZy families, we found that these families typically contain evidence of lineage specific duplications in the CAsc and CGsc species, as well as evidence of extensive gene loss in the other species.

*Colletotrichum* pathogens, including members of the CAsc, alkalinize the host tissue during their infection [54, 55]. Different protease families often favor different pH ranges. The two most overrepresented, metallo and serine peptidases (Fig. 3b), are most often considered highly active under alkaline or neutral conditions. Interestingly, secreted serine proteases have been shown to play a central role in both pathogenic and mutualistic relationships and are putatively involved in cell wall degradation [56–58]. However, aspartic proteases make up the third most expanded protease gene family, which are optimally active under acidic conditions such as the environments of the most common fruits it infects (e.g. strawberry, blueberry, apple, citrus, etc.). These data suggest that CAsc are equipped with proteases for various pH environmental conditions and perhaps important to certain stages of the infection.

All of the CAsc genomes contain a member of the plant-like subtilisin family (CFIO01_03718; CNYM01_02854; CSAL01_01351; CSIM01_08242) described by Armijos Jaramillo et al. [59, 60] as originating by a horizontal gene transfer event (HGT) and postulated to have a role in pathogenicity in the *C. graminicola*/maize interaction. This evidence reinforces the hypothesis that the HGT event occurred in an ancestor of the *Colletotrichum* lineage and was subsequently maintained, though natural selection in all extant species.

### Necrosis and ethylene-inducing peptide 1 (Nep1)-like proteins (NLPs)

**Fig. 4:**
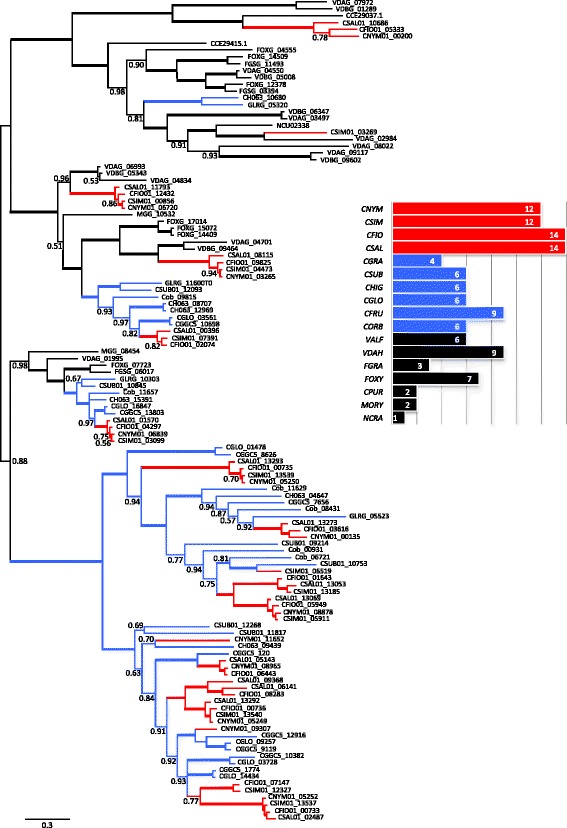
Phylogenetic reconstruction of secreted necrosis and ethylene-inducing peptide 1 (Nep1)-like proteins (NLPs). *Blue* branches highlight gene family expansions in *Colletotrichum acutatum species complex* and *green* branches expansions in other *Colletotrichum* species. The *bar* diagram shows the overall numbers of putative secreted NLPs identified in the genomes used in this study

NLPs trigger leaf necrosis and immunity associated responses exclusively in dicotyledonous plants [61]. NLPs are effectors that boost pathogen virulence during host colonization by disintegration of the plasma membrane. We identified NLPs by searching for the conserved motif GHRHDWE [61] in a six-frame translation of the genome sequences. In addition, we searched for proteins with the IPR term IPR008701. NLPs were particularly overrepresented in the CAsc and all four species have nearly twice as many NLPs as the other *Colletotrichum* spp. and other species included in this study. A phylogenetic reconstruction shows that most of the *Colletotrichum* spp. NLPs form a single clade that shows a rapid expansion of the gene family within *Colletotrichum* and three lineages of CAsc specific gene duplications (Fig. 4).

### Lineage Specific Effector Candidates (LSECs)

**Fig. 5:**
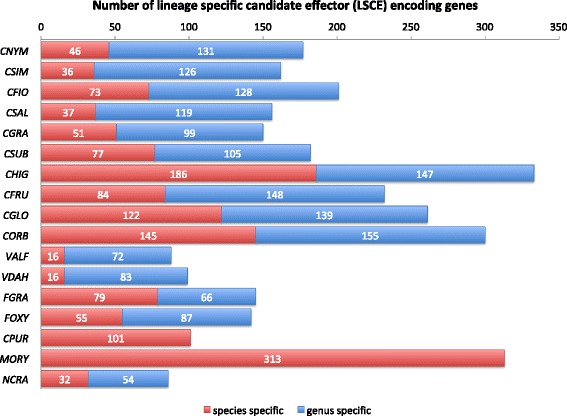
Lineage Specific Effector protein Candidates (LSECs) identified in *Colletotrichum* and other fungal genomes used in this study, based on no-BLAST sequence similarity with proteins predicted in other species (*red*) or other genera (*green*)

LSECs were identified as secreted proteins that have no homology to any other protein (species-specific) or that have homology to proteins from other members of the same genus (genus-specific). The predicted LSECs (Fig. 5) share properties that are consistent with known fungal protein effectors. They are small proteins, having an average length of 191 amino acids over all of the species analyzed whereas the average length of all proteins is 460 amino acids. In addition, they are cysteine-rich, with cysteine making up 2.43 % of the amino acid content. In LSECs of less than 200 amino acids, the cysteine content is even more pronounced, with those proteins having 3.38 % cysteine content and LSECs less than 100 amino acids in length having 5.6 %. An average of 174 LSECs were identified in the CAsc genomes, of those 48 have been identified as species-specific and 126 as genus-specific. Variation within the species complex is evident with *C. fioriniae* encoding the highest number: 201 LSECs (128 genus-specific and 73 species-specific) and *C. salicis* the lowest number: 156 LSECs (119 genus-specific and 37 species-specific).

The definitions of species-specific and genus-specific LSECs are sensitive to the presence or absence of closely related species in the GenBank as is demonstrated by the lack of genus-specific LSECs in *Magnaporthe oryzae* and *Claviceps purpurea* (Fig. 5). No additional annotated genomes of *Magnaporthe* or *Claviceps* spp. were available in GenBank at the time this study was completed. However, since all member of the same genus have approximately the same evolutionary distance to other genera, we reasoned that within species comparisons of the overall numbers of LSECs among different *Colletotrichum* spp. should reflect their relative importance of LSECs in each species. The LSECs content of the CAsc genomes are comparable to the content of the *C. graminicola* (150) and *C. sublineola* (182) genomes but are much lower than *C. higginsianum* (333), the *Colletotrichum* genome with the highest number of LSECs.

### Secondary metabolite synthesis capacity

**Fig. 6:**
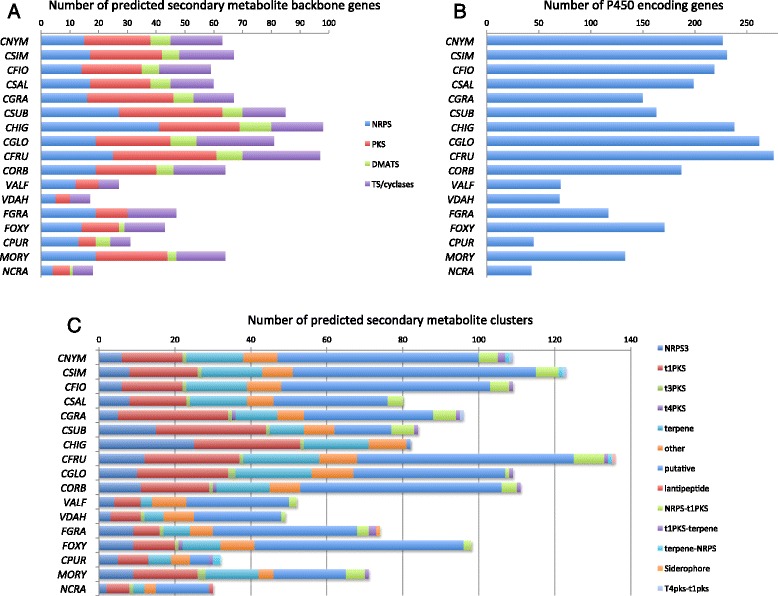
**a** Secondary metabolite-related backbones genes including non-ribosomal peptide synthetases (NRPS), polyketide synthases (PKS), DMATS-family aromatic prenyltransferases (DMATS), and terpene synthases/cyclases (TS) identified in the fungal genomes used in this study. **b** Cytochrome P450 monooxygenases genes identified in *Colletotrichum* species and the other fungal genomes used in this study. **c**. Secondary metabolite clusters predicted by antiSMASH [48] in *Colletotrichum* and the other fungal genomes used in this study

Fungi produce an enormous array of secondary metabolites, which may serve as signalling molecules and toxins against microorganisms (antimicrobials), plants (phytotoxins) or animals and humans (mycotoxins) [62, 63]. Precursor genes are required for the biosynthesis of SMs and are often located inside fungal gene clusters. In addition, tailoring enzymes such as cytochrome P450 monooxygenases (P450s), also located inside the gene clusters, form the final products [64]. *Colletotrichum* spp. have a large diversity of putative SM-related genes (Fig. 6a and b) and biosynthetic gene clusters (Fig. 6c and Additional file 7: Table S5) compared to other plant pathogenic fungi and thus hold the genetic potential to generate diverse SMs. The P450s are overrepresented in several *Colletotrichum* spp. (Fig. 6b).

Putative polyketide synthase (PKS) clusters and “backbone” genes within *Colletotrichum* spp. and *M. oryzae* are found in substantially higher numbers than in other ascomycetes (Fig. 6a). The PKS genes of *Colletotrichum* spp. are highly active and important to appressorium-mediated host penetration [17, 65–67]. The type 1 PKSs commonly responsible for the biosynthesis of macrolides are one of most abundant clusters within CAsc*.* and other *Colletotrichum* spp.

The number of terpenoid synthases and relative gene clusters in members of the CAsc are comparable to the other *Colletotrichum* spp. (Fig. 6a). Neither natural products nor hybrid gene clusters with terpenoid and NRPS origin are commonly seen in nature, but are predicted in the anti-SMASH analysis and only in *C. nymphaeae* and *C. simondsii*. In addition, the more common hybrid gene cluster t1PKS-terpene (e.g. meroterpenoids) was found in 7 out of 10 *Colletotrichum* spp. genomes. The terpenoid clusters and their PKS hybrids are potential candidates for synthesizing respectively, antimicrobial triterpenoids (ergosterols and derivatives) and phytotoxic meroterpenoids (i.e. colletotrichin and derivatives) by different *Colletotrichum* spp. [68–70].

Higher numbers of non-ribosomal peptide synthase (NRPS) genes and clusters are found within species of the genus *Colletotrichum* (Fig. 6a). Candidate genes and clusters potentially involved in the production of phytotoxic ferricrocin (e.g. siderophore) [71] and different diketopiperazines with phytotoxic and antimicrobial activities [72, 73] secreted by *C. gloeosporioides* are found within *Colletotrichum* spp. genomes. The non-ribosomal peptide mycosporine biosynthetic cluster has been characterized in fungi as well as other organisms [74]. Although *Colletotrichum* spp. have been shown to produce mycosporine with antimicrobial and phytotoxic activities [75, 76] no mycosporine biosynthetic clusters were identified in *Colletotrichum* spp. genomes in this work.

The precursor dimethylallyl tryptophan synthases (DMATS) within *Colletotrichum spp.* are found in lower numbers compared to the other precursors (Fig. 6a) but are overrepresented in *Colletotrichum* spp.

## Discussion

The availability of genome sequences of six species of *Colletotrichum* with differing host ranges and belonging to five different species complexes provided an opportunity to study the evolution of host range in the genus. We sequenced the genomes of four species belonging to the CAsc, a group of typically broad host range pathogens and compared gene content among all of the genomes. In order to investigate changes in gene content between host-specific and broad host range pathogens we focus our study on two classes of molecules known to have a direct role in fungus/plant interaction. We characterized genes involved in secondary metabolite biosynthesis and genes encoding secreted proteins. Generally, pathogens characterized by a hemibiotrophic lifestyle such as *Colletotrichum* spp. and *Magnaporthe oryzae,* secrete a higher percentage of proteins extracellularly [18, 77]. Hierarchical clustering of gene family and functional domain assignments revealed overrepresentation of CAZy and protease gene families with both the CAsc and CGsc with respect to the other fungal genomes analyzed. Members of these species complexes are broad host range pathogens, suggesting that the higher number in CAZy and protease diversity may be associated with the ability to infect multiple host species. Hierarchical clustering of the 13 studied species resulted in a cluster comprised of CAsc and CGsc species, two distantly related species complexes. This result highlights the similarity in both secretomes and whole proteomes of these species complexes and suggests that their gene family content, especially their repertoires of CAZymes and peptidases are the product of recent, lineage specific expansions of these families independently in each species complex. Interestingly, phylogenetic analyses of the CAZyme and peptidase families revealed that, in contrast to our expectations, gene loss in other *Colletotrichum* species is as important, if not more important force driving the evolution of gene family size.

A recent work focused on *Colletotrichum* comparative genome analyses [78] reported that the content secreted proteins, CAZy and proteases of members from the CAsc and CGsc were more similar to each other despite their phylogenetic distance. Analyses carried out by these authors reveal that *Colletotrichum* species have tailored profiles of their carbohydrate-degrading enzymes according to their infection lifestyles. Phylogenetic analyses revealed lineage-specific expansions of GH43 and S10 members within the CAsc and CGsc, with duplications of specific genes within the respective lineages. However, close examination of the phylogenies of Gan et al. [78] reveals gene losses in lineages outside of the CAsc and CGsc, providing further support of the view that gene loss, in addition to gene duplication is an important component of the adaptation of gene families.

The comparative analysis of fungal secretomes reported by Zhao et al. [14] revealed that plant pathogens typically have higher numbers of CAZymes than non-pathogens and that biotrophic fungi have a lower diversity of CAZymes than do necrotrophs and hemibiotrophs. The same work showed that there were similar fractions of each CAZyme class found in pathogens that infect similar hosts, such as monocots or dicots. This evidence confirms what has been revealed by the genome comparison between *C. graminicola* and *C. higginsianum* by O’Connell et al. [17]. Comparative analyses of 18 fungi with diverse pathogenic lifestyles and strategies [56] revealed that the overall distribution of genes encoding CAZyme involved in plant cell wall breakdown reflect the different taxonomic groups, and that it may in fact reflect differences in cellulose, xylan and pectin of the host plants. A more recent work focused on comparative analysis of 79 fungal genomes [79] revealed that more CAZymes were found to be specific to each species compared with the Magnaporthaceae specific clusters suggesting that CAZyme gene sequences are plastic and may contribute to speciation. Furthermore the authors hypothesize that in the Magnaporthaceae CAZymes may vary based on route of infection rather than the type of host plant [79]. These results are consistent with the idea that different lifestyles, hosts and host tissues present different types of carbohydrate substrates to the pathogen this is reflected by each species’ CAZyme repertoire. However to investigate the evolutionary processes associated with gene expansions/losses research needs to focus on closely related species (or isolates belonging to the same species) with different lifestyles, hosts and host specificities.

The CAsc and CGsc genomes have the highest number of fungal CAZymes involved in the degradation of plant cell walls, such as xyloglucan, xylan, pectin and cellulose [80, 81]. CAsc and CGsc are also characterized by the broadest arsenal of secreted peptidases such as metallo and serine peptidases, both families with known roles in plant pathogenesis [82–84]. These proteases may contribute to the degradation of the plant cell wall by targeting structural proteins. Changes in gene families encoding for degradative enzymes reflect the evolutionary adaptation of species complexes to different hosts and niches and open new opportunities for the identification of novel genes for industrial purposes.

Examination of protein domain and family content using InterPro revealed changes in NLPs with CAsc carrying twice the number of the genes compared to other fungi. NLPs trigger leaf necrosis and immunity associated responses exclusively in dicotyledonous plants [85]. In *C. higginsianum* the cell death inducer ChNLP1 is upregulated during the transition from biotrophy to necrotrophy. Intriguingly, Kleemann et al. [86] have also found some effectors (ChECs) that suppressed cell death induced by ChNLP1 during the initial biotrophic phase, in order to maintain cell viability. The remarkable repertoire of NLPs found in the CAsc might reflect their ability to infect a wide range of hosts. Some of these proteins may function as necrosis inducing proteins, whereas other NLPs may have roles in overcoming early plant defense responses [86].

Many NLPs have been characterized; however, only a few from the genomes used in this study have been shown to induce necrosis in plant. Since most of the previous work has focused on NLPs characterization under laboratory conditions, the hypothesis is that they might have different role in different hosts and/or they might act at different conditions providing “flexibility” to the pathogen. Considering the high number of NLPs encoded by CAsc it could be used as model system to study NLP evolution and their biological role. This study also revealed lineage specific contractions of LSECs and expansions of NLP families within the CAsc, LSECs are important for suppressing the host immune system, enabling pathogens to colonize host tissues [87–89]. Both *C. graminicola* and *C. sublineola* also have reduced numbers of LSECs but the expansion of NLPs is only found in the CAsc indicating that it may be a lineage specific innovation that is unique to members of the CAsc.

*Colletotrichum* spp. have a large arsenal of putative SM-related backbone genes, clusters and tailoring enzymes compared to other plant pathogenic fungi and thus hold the genetic potential to generate diverse SMs. P450s play central roles in the detoxification of plant-derived antimicrobials [90] and in the biotransformation of industrial-related products [64]. The large diversity of P450s identified in *Colletotrichum* spp. may explain how these pathogens detoxify phytoalexins [91] and why these fungi have been investigated for their capacity to biotransform various products (e.g. terpenoids) [92]. The PKS genes and clusters are most abundant within *Colletotrichum* spp. PKSs are important to host penetration and are commonly responsible for macrolides biosynthesis. This is of great interest, as *Colletotrichum* spp. can synthesize various macrolides (e.g. monocillins I-III, monoorden, colletodiol, etc.) with phytotoxic and antimicrobial activities including novel macrolides produced by CAsc [93–96]*.* DMATS are overrepresented in *Colletotrichum* spp. and in those pathogens characterized by a biotrophic stage. Genome-wide expression profiles of *Colletotrichum* spp. reveals low activity of DMATS [17, 67] with few highly active of *C. orbiculare* during plant infection [65]. Secretion of phytotoxic alkaloids by *Colletotrichum* spp. with importance to plant infection has not been found, but identified gene cluster in genome sequencing are potential candidates to different compounds with anticancer and antimicrobial activities among others found within this genus [97–99].

Earlier studies concluded that gene family content in fungi depends more on evolutionary lineage, and suggested that lifestyle is less important in driving changes in gene family size [13]. In this study we focused on one genus and compared several species belonging to species complexes that differ in host range. We compared the gene content of several members of the genus *Colletotrichum* and found that changes in several gene families, particularly CAZymes and proteases are associated with two distantly related lineages of *Colletotrichum* spp. that have broad host range.

## Conclusions

In this manuscript, we provide an analysis of adaptations in gene content that are associated with broad host range in *Colletotrichum* spp. This study illustrates the plasticity of fungal genomes, and shows that relatively recent changes in gene content are associated with major changes in host range. It is intriguing to speculate a cause-effect relationship between a decrease in CAZyme and protease diversity and decrease in host range. Future studies of additional taxa both within and outside of the genus *Colletotrichum* will show whether lifestyle changes drive gene duplication and loss in all fungi and whether CAZymes and peptidases are the key families controlling host range. This study also demonstrates the need for higher resolution taxonomic sampling in order to better understand the role of gene duplication and loss in the evolution of fungal genomes and the possible association with biological characters.

## Additional files

Additional file 1: Table S1. List of *Colletotrichum* strains used for the phylogeny shown in Additional file 2: Figure S1, indicating their species designation, complex, strain ID and Genbank accession numbers of the sequences retrieved and used for phylogenetic analyses. Strains included in genome sequence projects are highlighted in bold. (XLSX 67 kb) Additional file 2: Figure S1. Phylogenetic analysis of the 133 *Colletotrichum* spp. listed in Additional file 1: Table S1 based on a multilocus concatenated alignment of the ITS, TUB2, ACT and GAPDH genes. A Markov Chain Monte Carlo (MCMC) algorithm was used to generate phylogenetic trees with Bayesian probabilities using MrBayes 3.2.1. The species complexes described by Cannon et al. [2] and Liu et al. [49] as well as *C. acutatum* species complex clades described by Damm et al. [5] are shown. *Monilochaetes infuscans* was used as an outgroup. (PDF 593 kb) Additional file 3: Table S2. InterPro functional domains associated with the proteomes and secretomes of fungal genomes used in this study. (XLSX 645 kb) Additional file 4: Table S3. Number of secreted CAZyme modules determined according to the CAZy database (http://www.cazy.org/) [50, 52]. (XLSX 21 kb) Additional file 5: Figures S2–S13. Figures S2 and S3. Clustering of secreted carbohydrate-active enzyme (S2) and peptidase (S3) families encoding genes identified in this study. Numbers of genes in each row were normalized using MeV 4.8.1. Hierarchical clustering of genes and species was performed and visualized using the package “pheatmap” 1.0.8 within R. Figures S4–S13. Phylogenetic trees of secreted proteins belonging to specific class of peptidases (Figure S4. A01A; Figure S5. S10; Figure S6. M43B; Figure S7. M35) and carbohydrate-active (Figure S8. AA3; Figure S9. AA7; Figure S10. CE10; Figure S11. CE16; Figure S12. GH5, Figure S13. GH43) enzyme families identified in the genomes analyzed in this study. Proteins were aligned with MAFFT and trees were inferred using the FastTree algorithm implemented in Geneious 8.1.4. Protein names in red are from CAsc species, Protein names in blue are from other *Colletotrichum* spp. (PDF 8509 kb) Additional file 6: Table S4. Numbers of extracellular secreted protease homologs classified according to the MEROPS database 10.0 [45] in *Colletotrichum* and other fungal genomes used in this study. (XLSX 14 kb) Additional file 7: Table S5. The secondary metabolite biosynthesis backbones genes and clusters in the four sequenced *Colletotrichum acutatum* species. Cluster were predicted by AntiSMASH version 1.1.2 [48]. BLASTp and RunIprScan were used to manually identify: nonribosomal peptide synthetases (NRPS; IPR010071, IPR006163, IPR001242), polyketide synthases (PKS; IPR013968), DMATS-family aromatic prenyltransferases (DMATS: IPR017795, Pfam PF11991), and terpene synthases/cyclases (TS: IPR008949) [47]. (XLSX 766 kb)
