# Supplementary material for: Gene family expansions and contractions are associated with host range in plant pathogens of the genus Colletotrichum
Source: BMC Genomics. 2016 Aug 5;17:555. doi: 10.1186/s12864-016-2917-6 (PMC4974774; doi:10.1186/s12864-016-2917-6)
Supplement: Additional file 2: Figure S1. — Phylogenetic analysis of the 133 Colletotrichum spp. listed in Additional file 1: Table S1 based on a multilocus concatenated alignment of the ITS, TUB2, ACT and GAPDH genes. A Markov Chain Monte Carlo (MCMC) algorithm was used to generate phylogenetic trees with Bayesian probabilities using MrBayes 3.2.1. The species complexes described by Cannon et al. [2] and Liu et al. [49] as well as C. acutatum species complex clades described by Damm et al. [5] are shown. Monilochaetes infuscans was used as an outgroup. (PDF 593 kb) [file 12864_2016_2917_MOESM2_ESM.pdf]

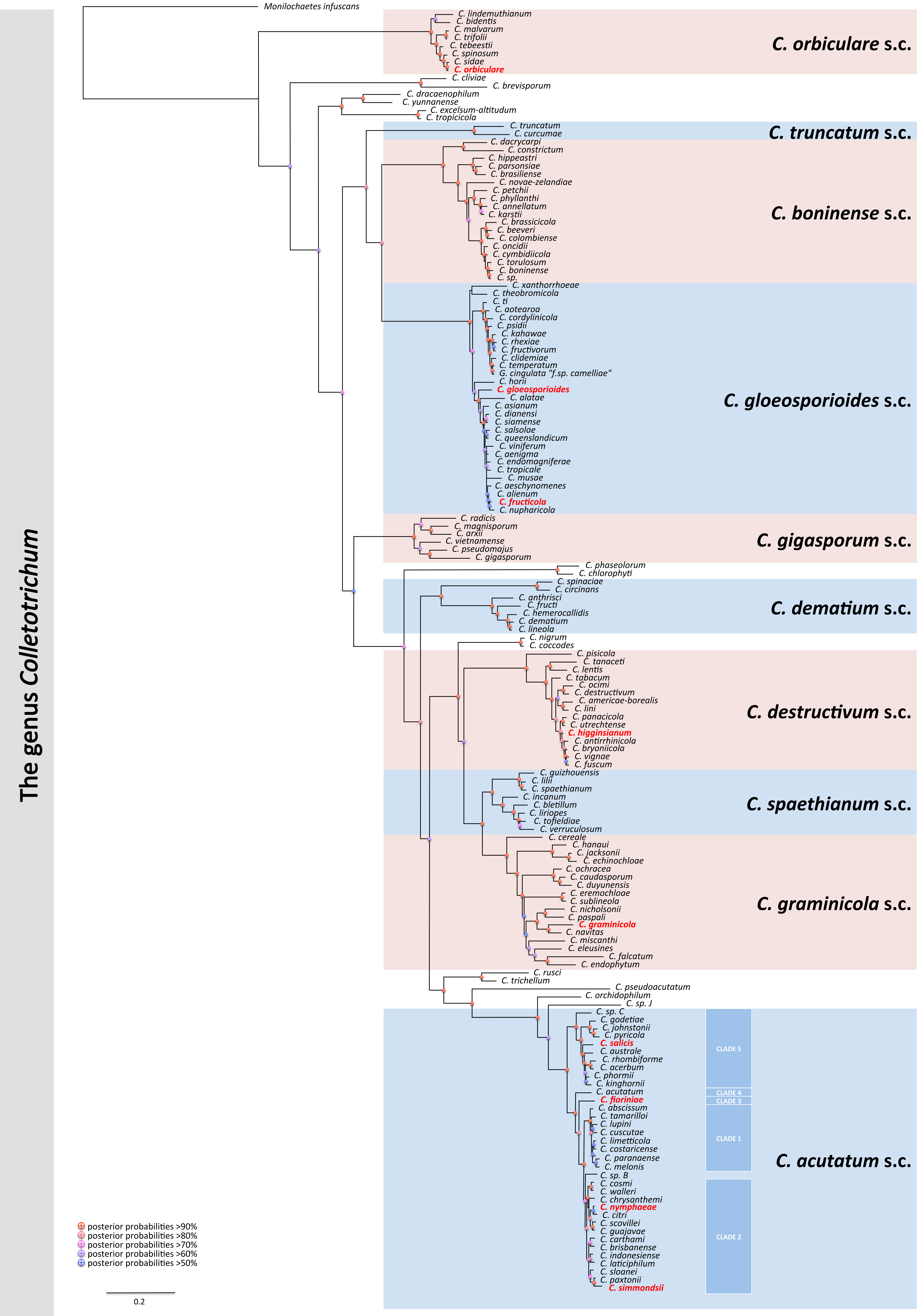
The genus Colletotrichum
Monilochaetes infuscans
C. lindemuthianum
C. bidentis
C. malvarum
C. trifolii
C. tebeestii
C. spinosum
C. sidae
C. orbiculare
C. orbiculare s.c.
C. cliviae
C. brevisporum
C. dracaenophilum
C. yunnanense
C. excelsum-altitudum
C. tropicicola
C. truncatum
C. curcumae
C. truncatum s.c.
C. dacrycarpi
C. constrictum
C. hippeastri
C. parsonsiae
C. brasiliense
C. novae-zelandiae
C. petchii
C. phyllanthi
C. annellatum
C. karstii
C. brassicicola
C. beeveri
C. colombiense
C. oncidii
C. cymbidiicola
C. torulosum
C. boninense
C. sp.
C. boninense s.c.
C. xanthorrhoeae
C. theobromicola
C. ti
C. aotearoa
C. cordylinicola
C. psidii
C. kahawae
C. rhexiae
C. fructivorum
C. clidemiae
C. temperatum
G. cingulata "f.sp. camelliae"
C. horii
C. gloeosporioides
C. alatae
C. asianum
C. dianensi
C. siamense
C. salsolae
C. queenslandicum
C. viniferum
C. aenigma
C. endomagniferae
C. tropicale
C. musae
C. aeschynomenes
C. alienum
C. fructicola
C. nupharicola
C. gloeosporioides s.c.
C. radicis
C. magnisporum
C. arxii
C. vietnamense
C. pseudomajus
C. gigasporum
C. gigasporum s.c.
C. phaseolorum
C. chlorophyti
C. spinaciae
C. circinans
C. anthrisci
C. fructi
C. hemerocallidis
C. dematium
C. lineola
C. dematium s.c.
C. nigrum
C. coccodes
C. pisicola
C. tanaceti
C. lentis
C. tabacum
C. ocimi
C. destructivum
C. americae-borealis
C. lini
C. panacicola
C. utrechtense
C. higginsianum
C. antirrhinicola
C. bryoniicola
C. vignae
C. fuscum
C. destructivum s.c.
C. guizhouensis
C. lilii
C. spaethianum
C. incanum
C. bletillum
C. liriopes
C. tofieldiae
C. verruculosum
C. spaethianum s.c.
C. cereale
C. hanaui
C. jacksonii
C. echinochloae
C. ochracea
C. caudasporum
C. duyunensis
C. eremochloae
C. sublineola
C. nicholsonii
C. paspali
C. graminicola
C. navitas
C. miscanthi
C. eleusines
C. falcatum
C. endophytum
C. graminicola s.c.
C. rusci
C. trichellum
C. pseudoacutatum
C. orchidophilum
C. sp. J
C. sp. C
C. godetiae
C. johnstonii
C. pyricola
C. salicis
C. australe
C. rhombiforme
C. acerbum
C. phormii
C. kinghornii
C. acutatum
C. fioriniae
C. abscissum
C. tamarilloi
C. lupini
C. cuscutae
C. limetticola
C. costaricense
C. paranaense
C. melonis
C. sp. B
C. cosmi
C. walleri
C. chrysanthemi
C. nymphaeae
C. citri
C. scovillei
C. guajavae
C. carthami
C. brisbanense
C. indonesiense
C. laticiphilum
C. sloanei
C. paxtonii
C. simmondsii
CLADE 5
CLADE 4
CLADE 3
CLADE 1
CLADE 2
C. acutatum s.c.
posterior probabilities >90%
posterior probabilities >80%
posterior probabilities >70%
posterior probabilities >60%
posterior probabilities >50%
0.2
