# Supplementary material for: Gene family expansions and contractions are associated with host range in plant pathogens of the genus Colletotrichum
Source: BMC Genomics. 2016 Aug 5;17:555. doi: 10.1186/s12864-016-2917-6 (PMC4974774; doi:10.1186/s12864-016-2917-6)

Figure S2 - Heatmap of secreted CAZY families

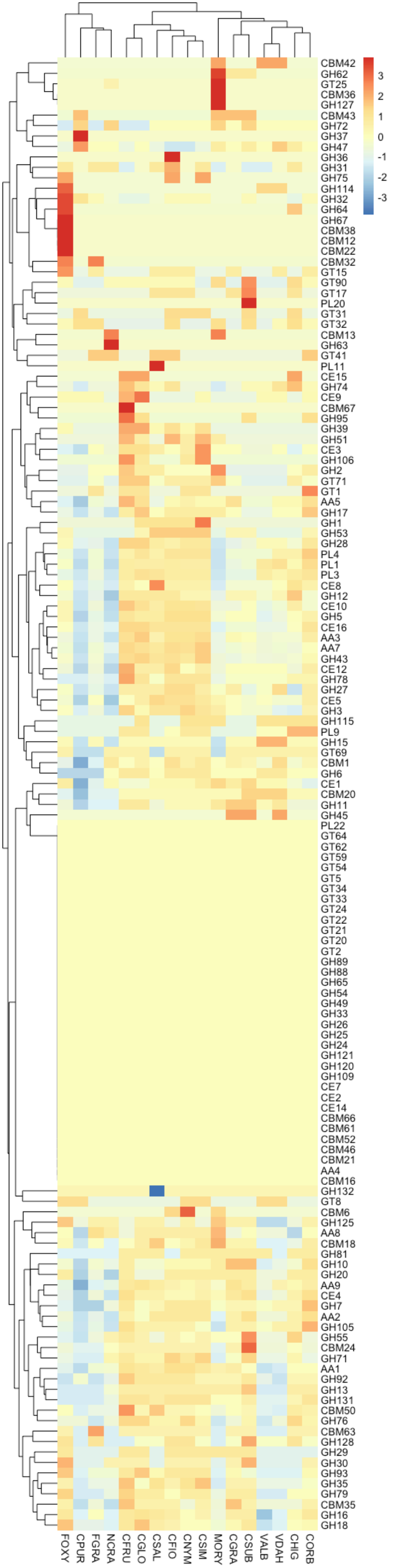

Figure S3 - Heatmap of secreted protease families

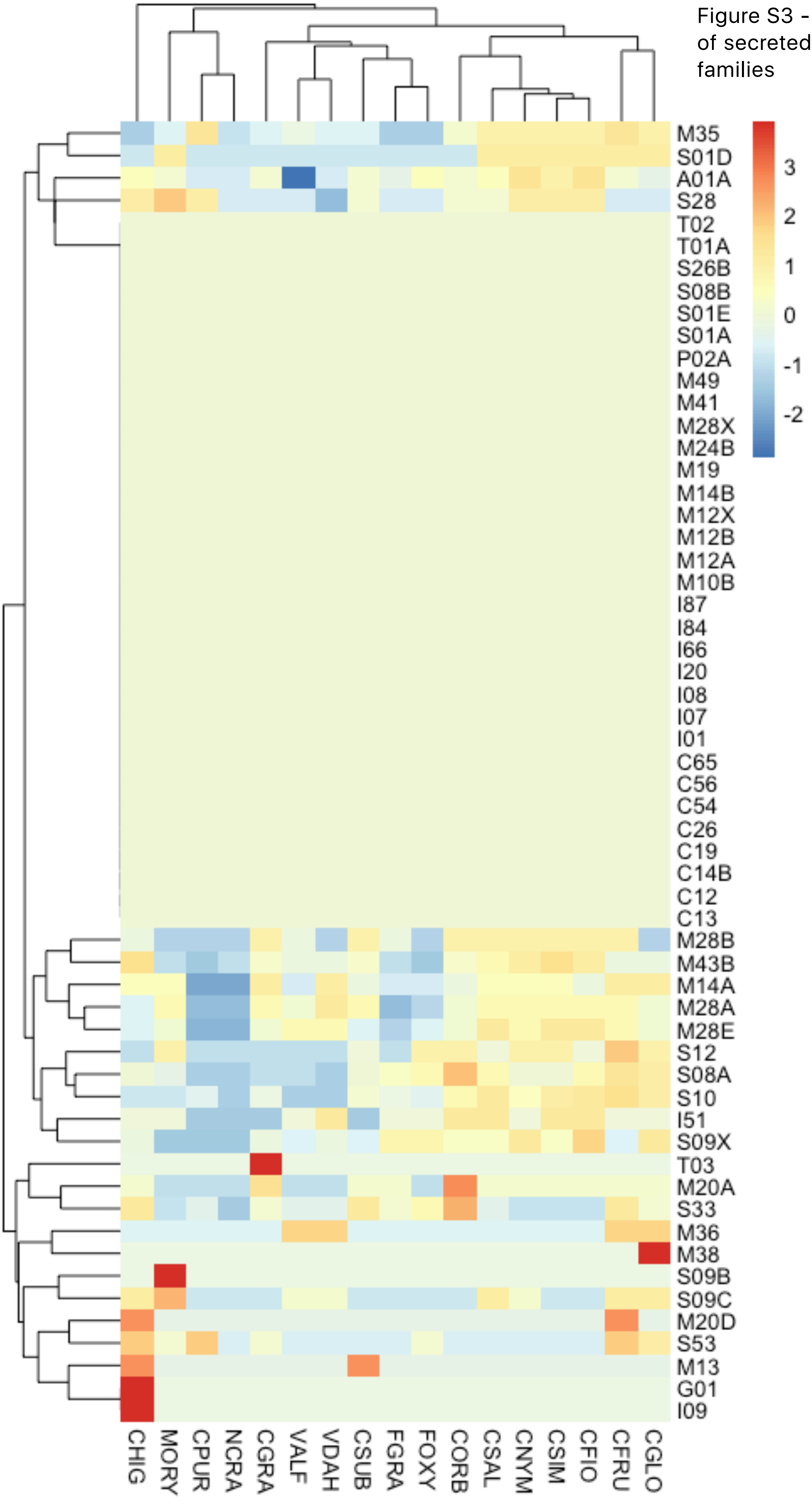

Figure S4 - Phylogenetic tree of secreted A01A peptidases

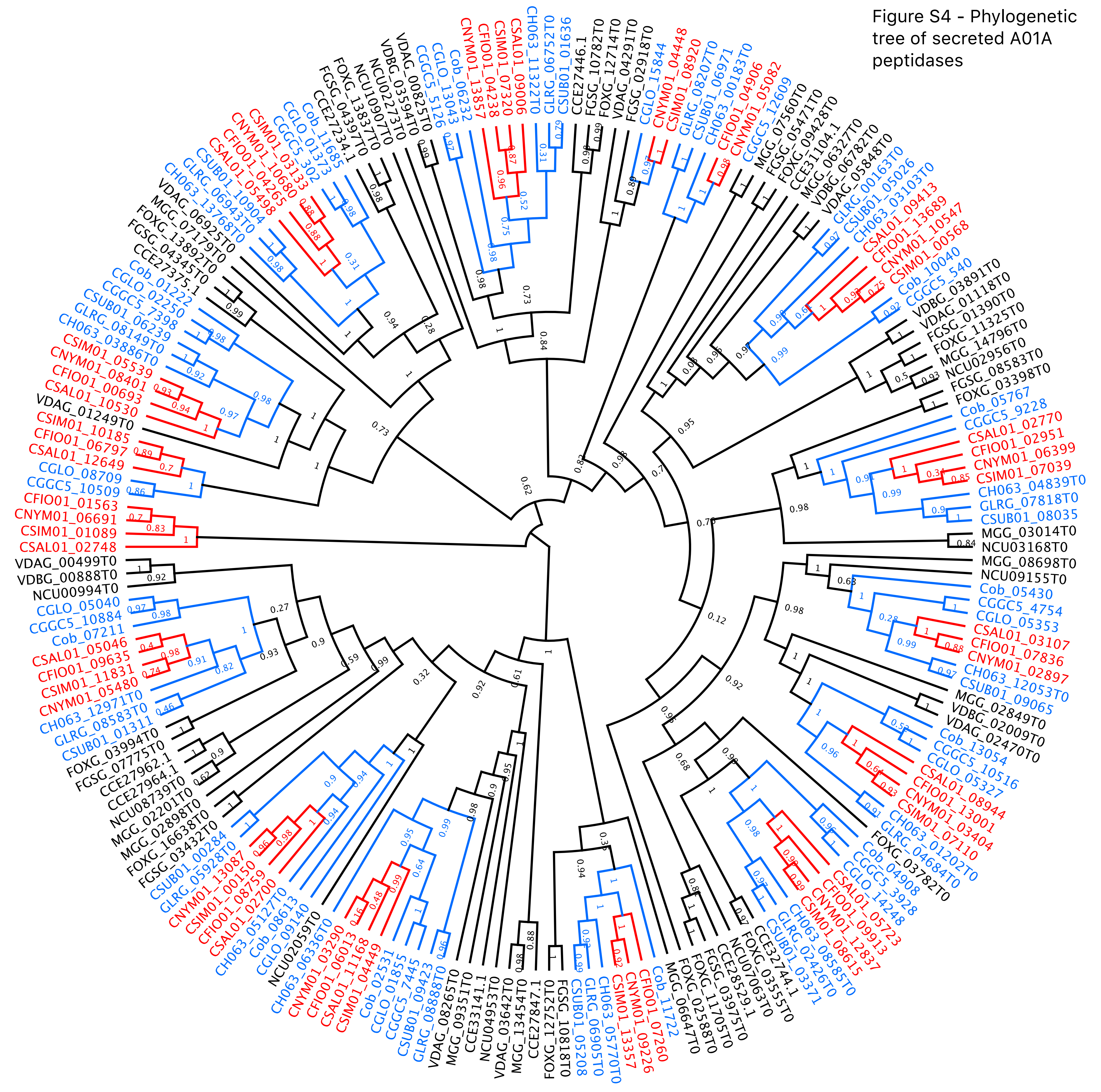

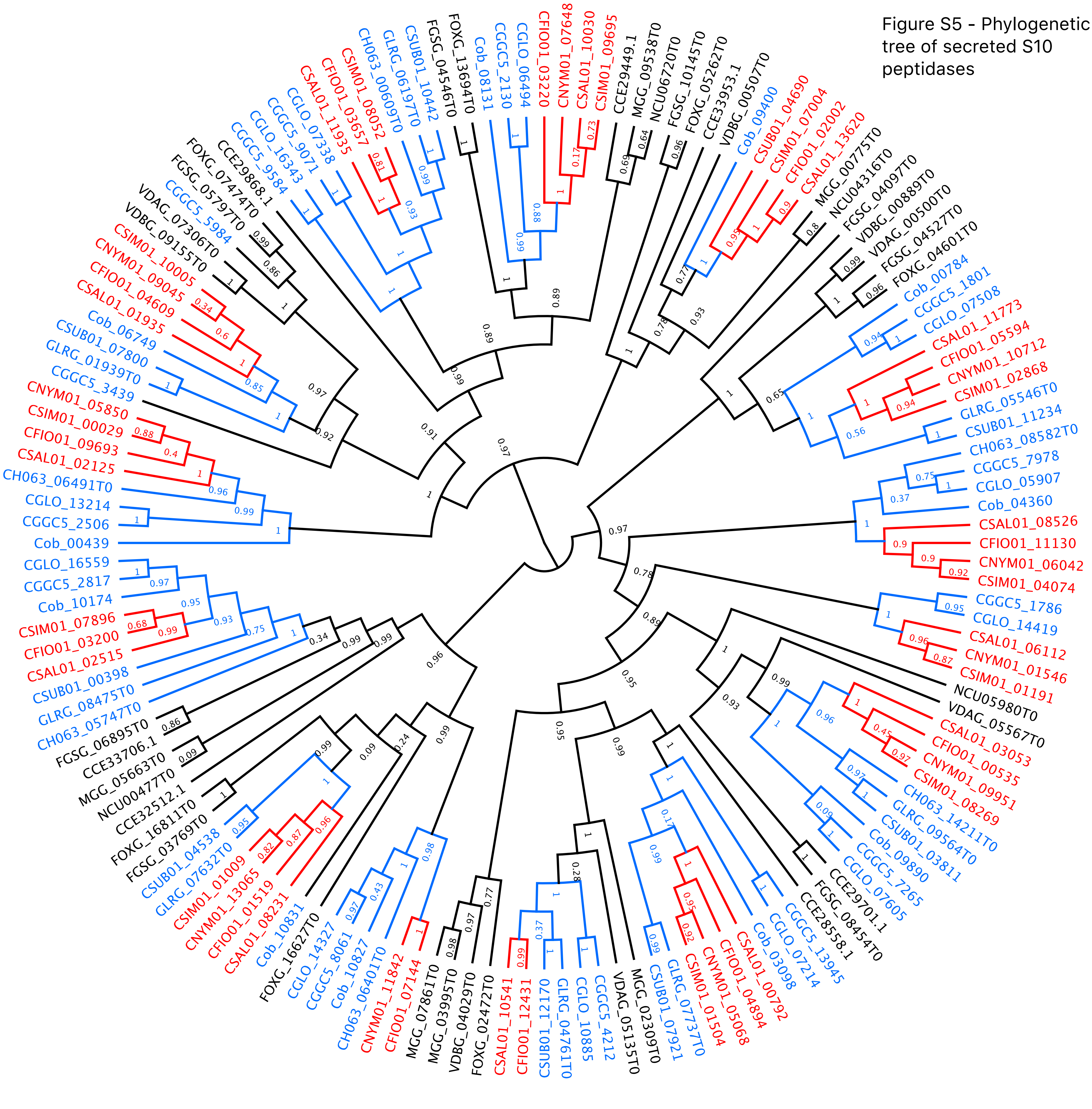

Figure S5 - Phylogenetic tree of secreted S10 peptidases

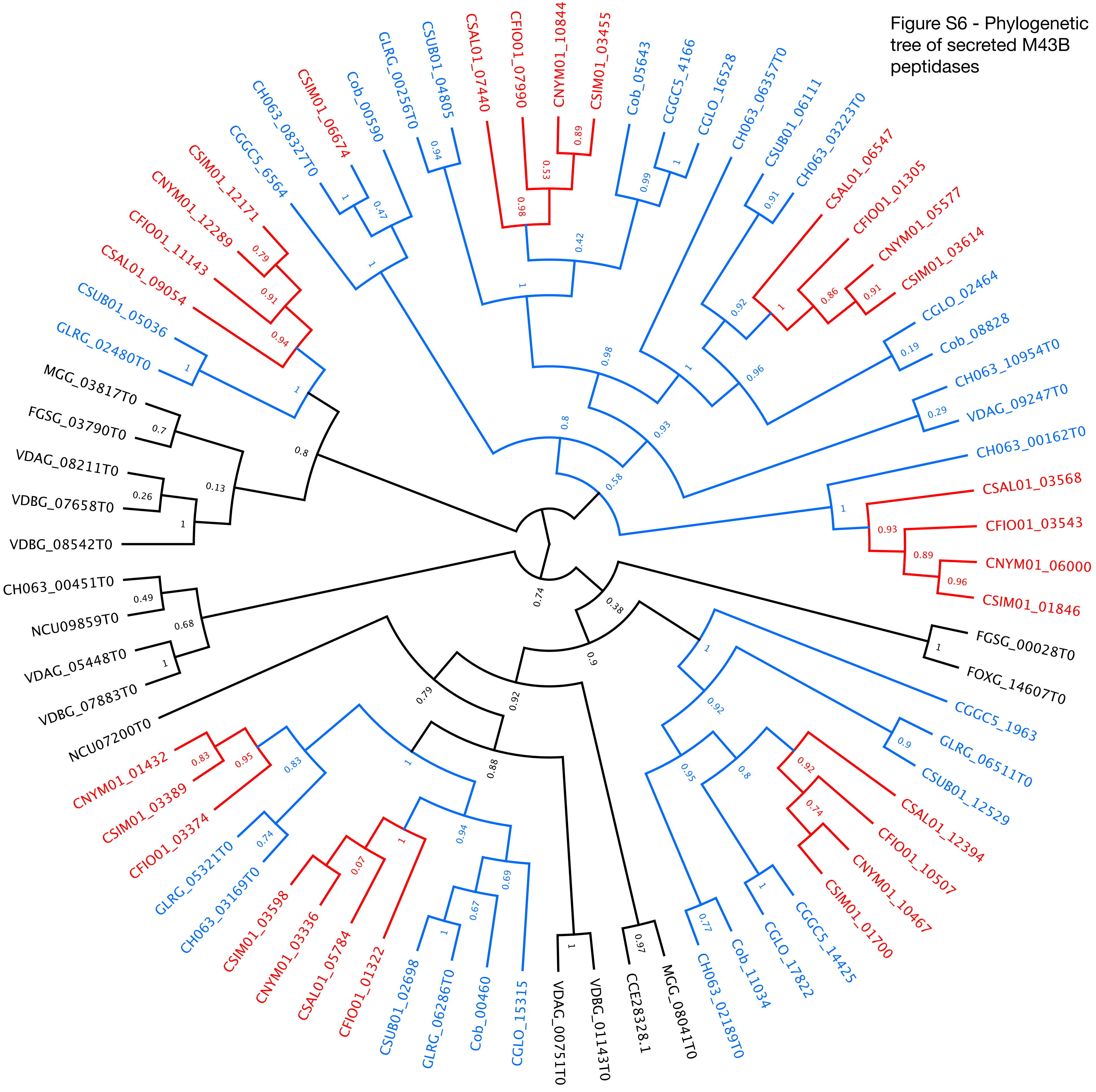

Figure S6 - Phylogenetic tree of secreted M43B peptidases

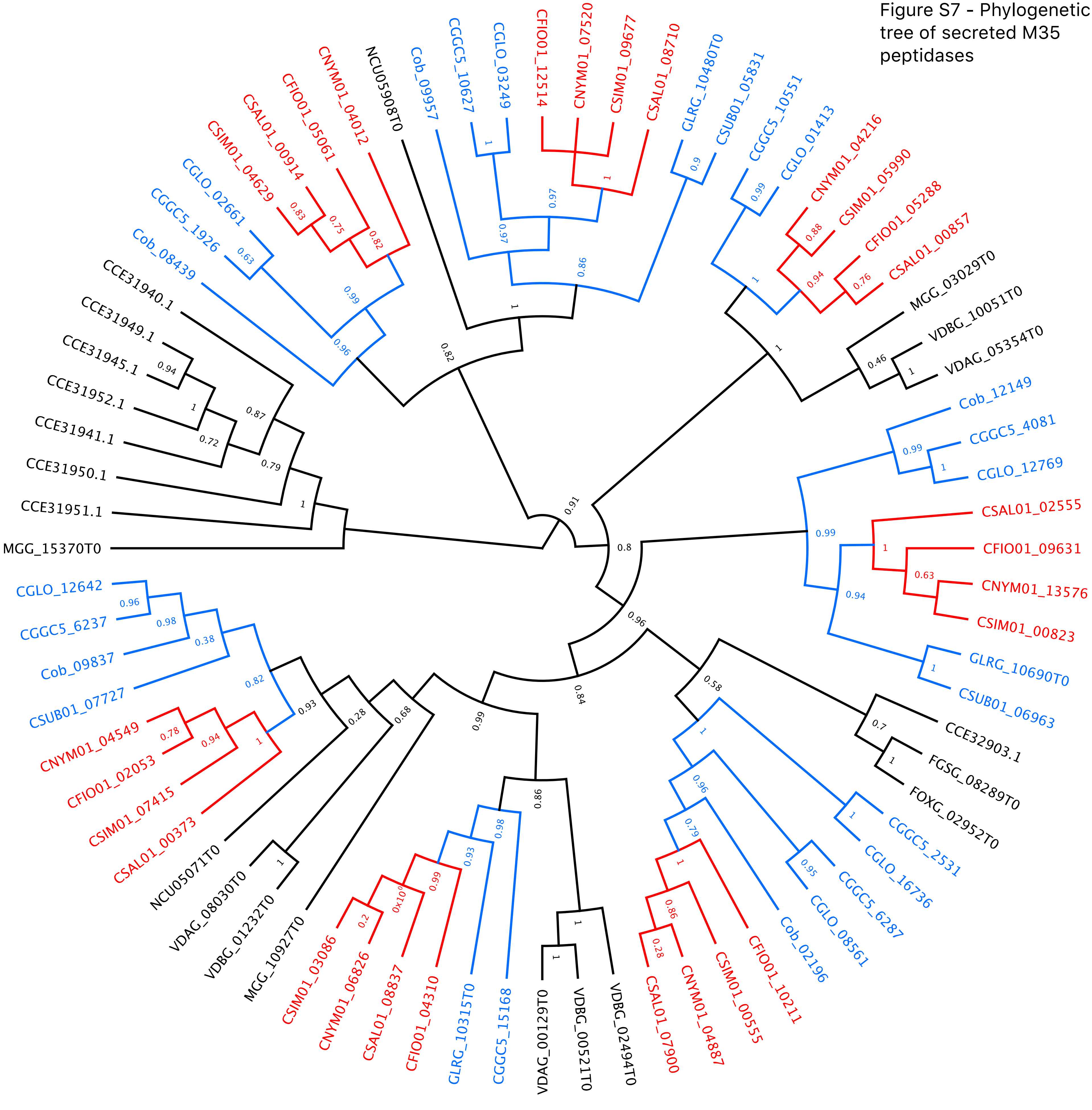

Figure S7 - Phylogenetic tree of secreted M35 peptidases

Figure S8 - Phylogenetic tree of secreted AA3 proteins

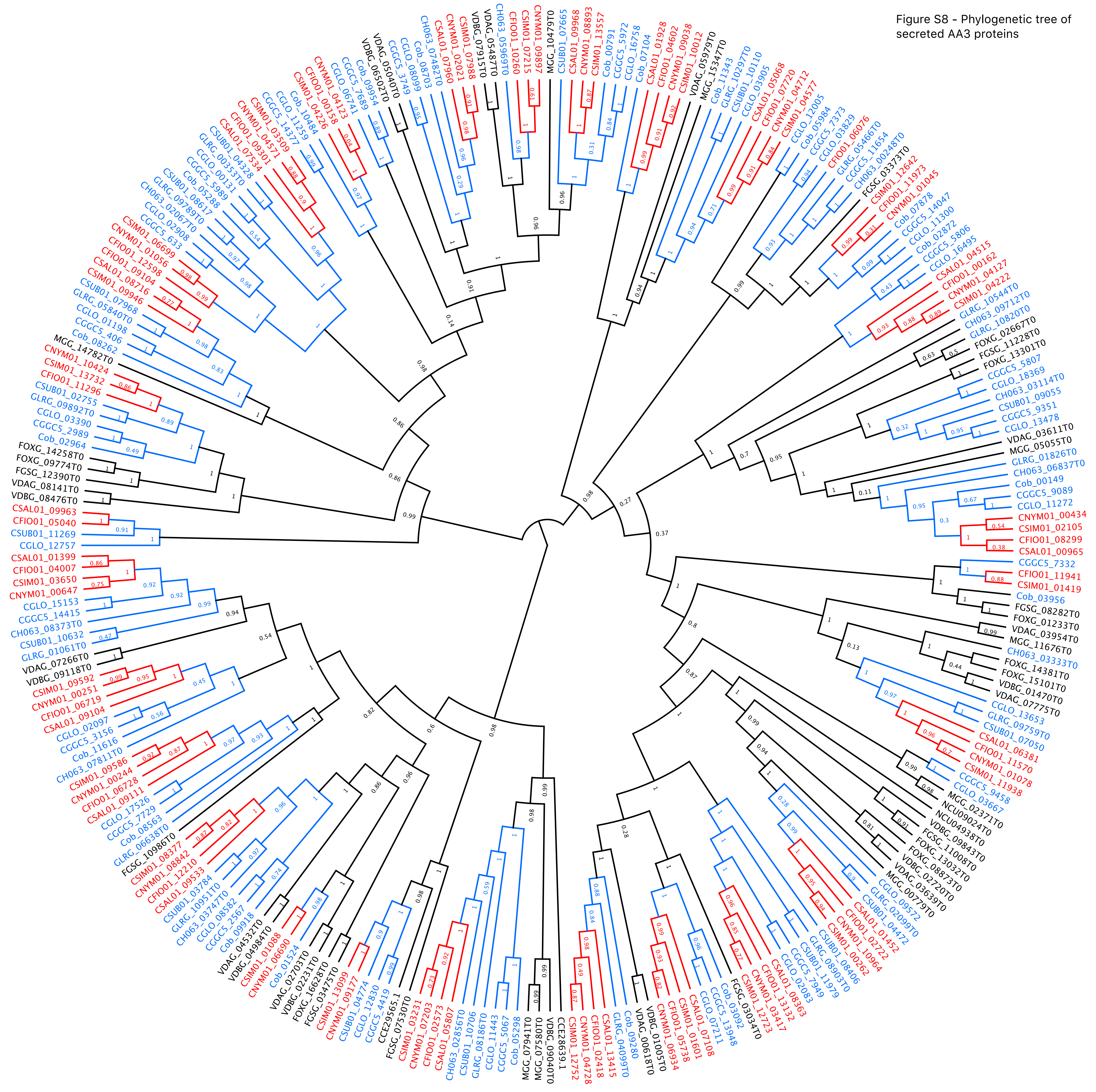

Figure S9 - Phylogenetic tree of secreted AA7 proteins

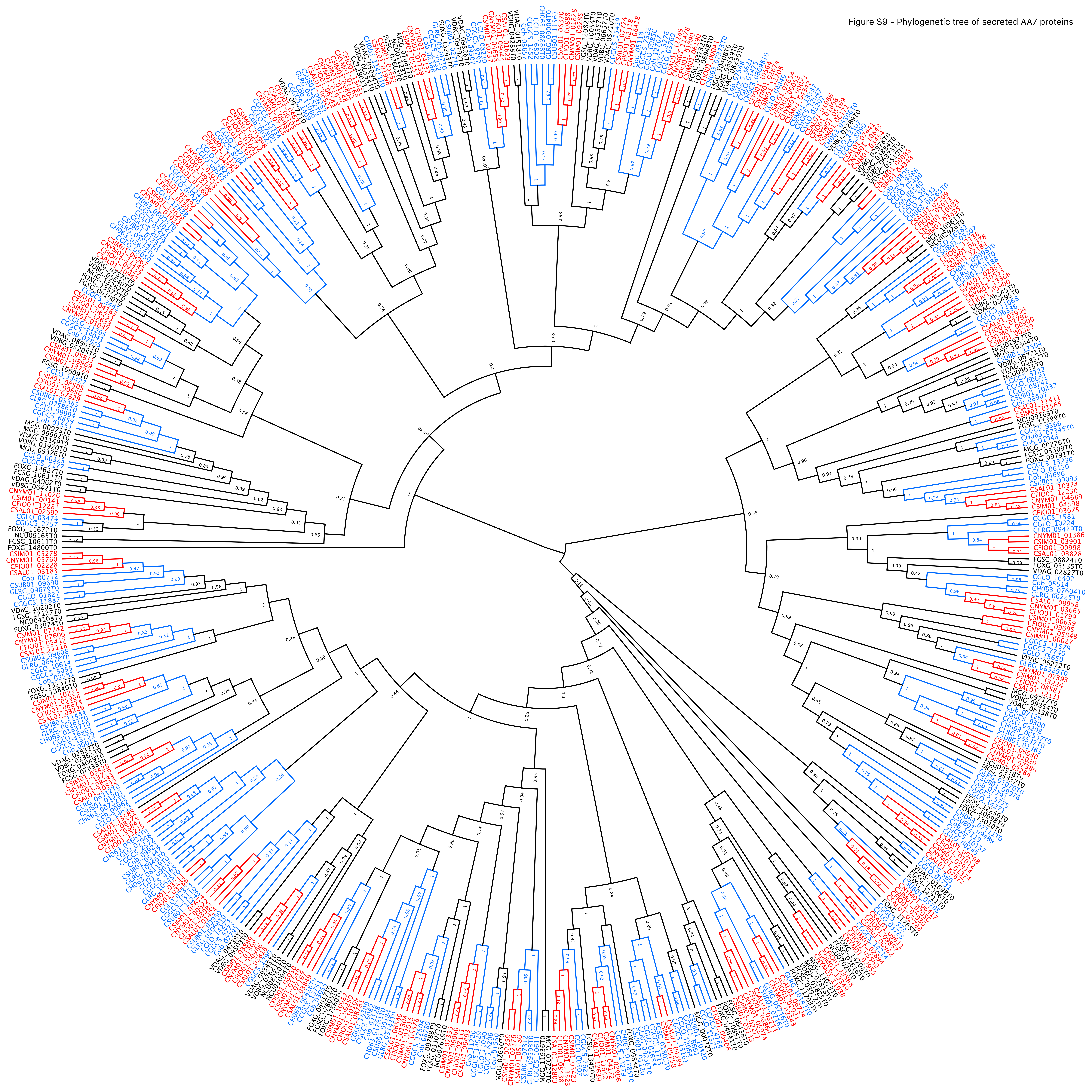

Figure S10 - Phylogenetic tree of secreted CE10 proteins

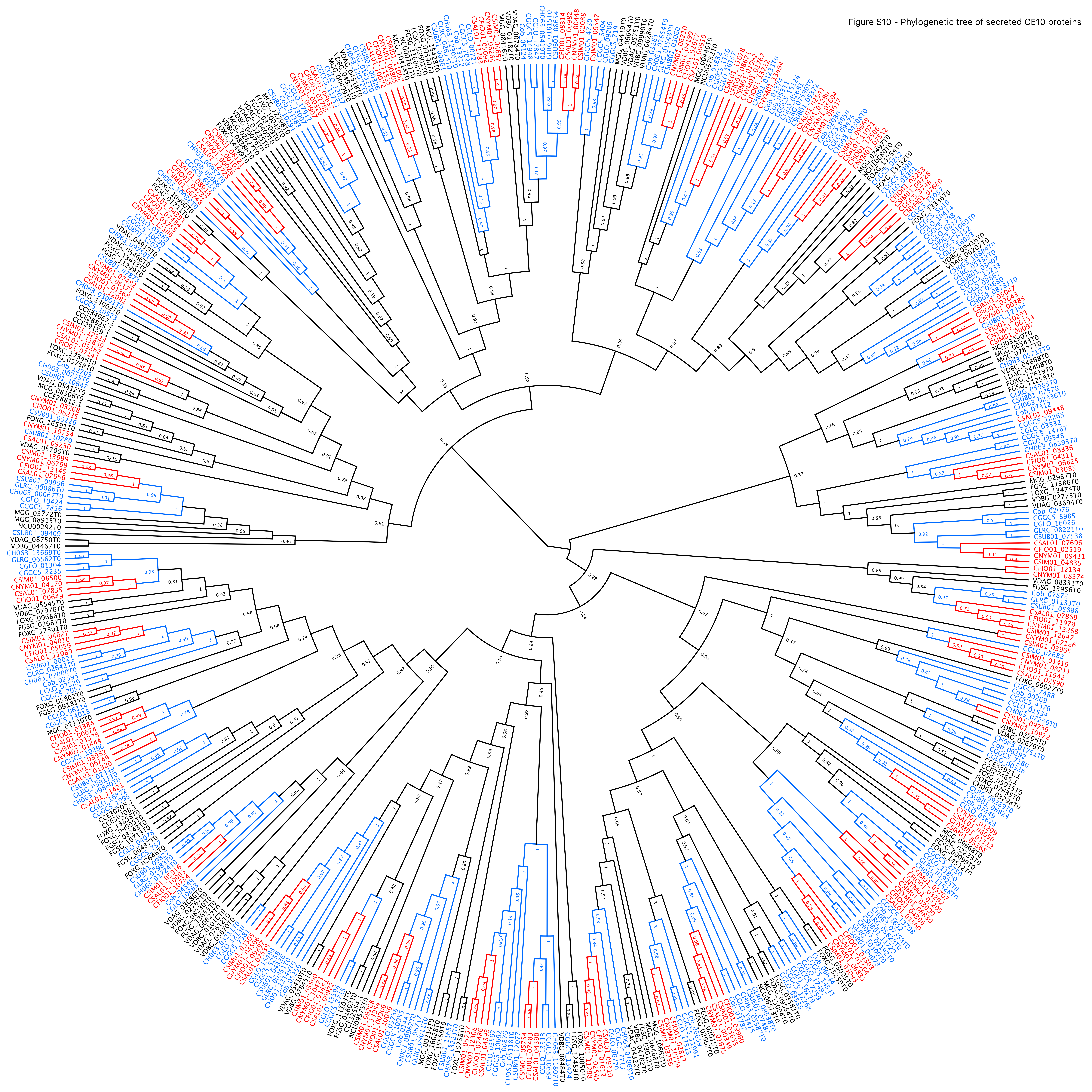

Figure S11 - Phylogenetic tree of secreted CE16 proteins

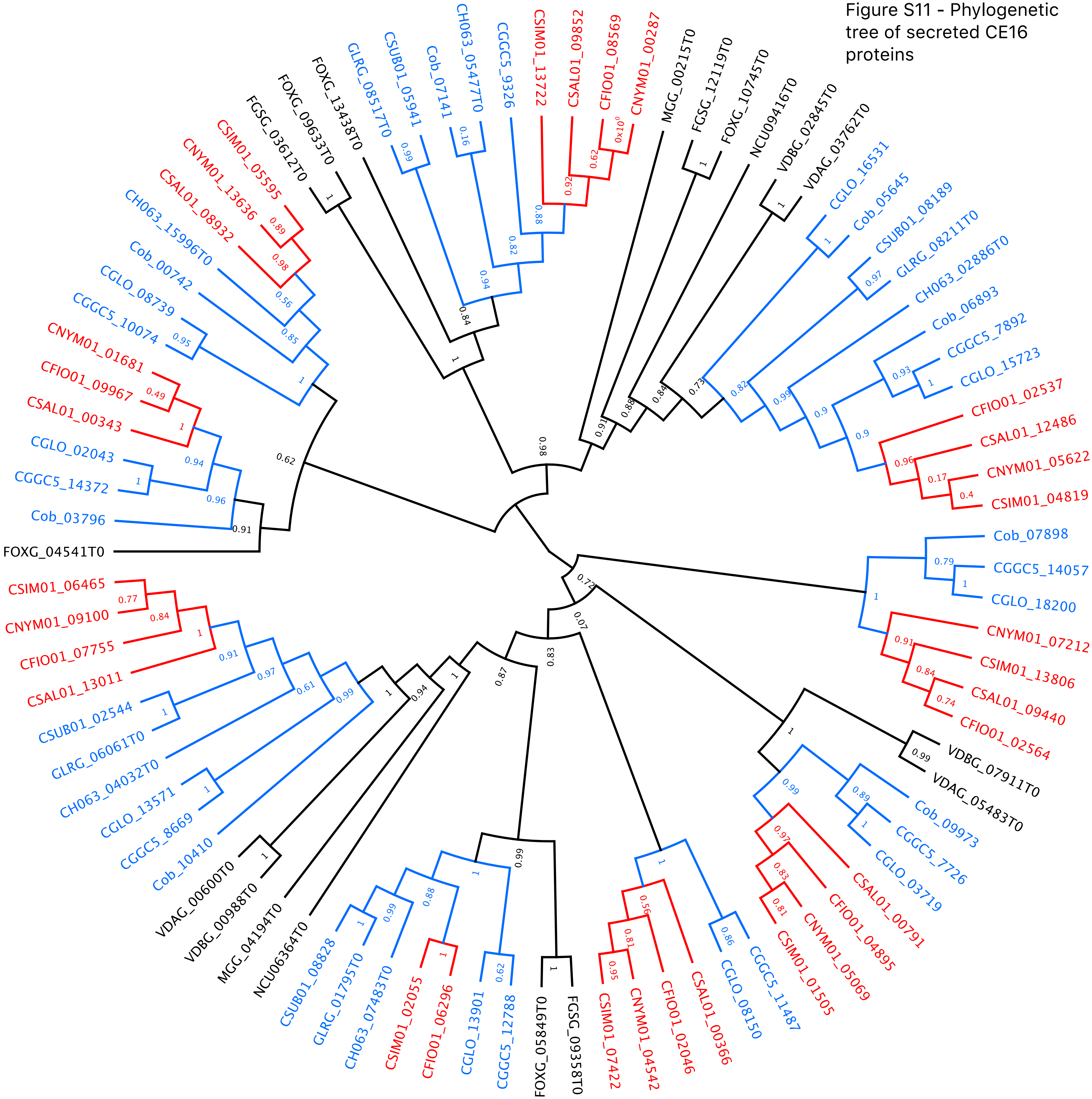

Figure S12 - Phylogenetic tree of secreted GH5 proteins

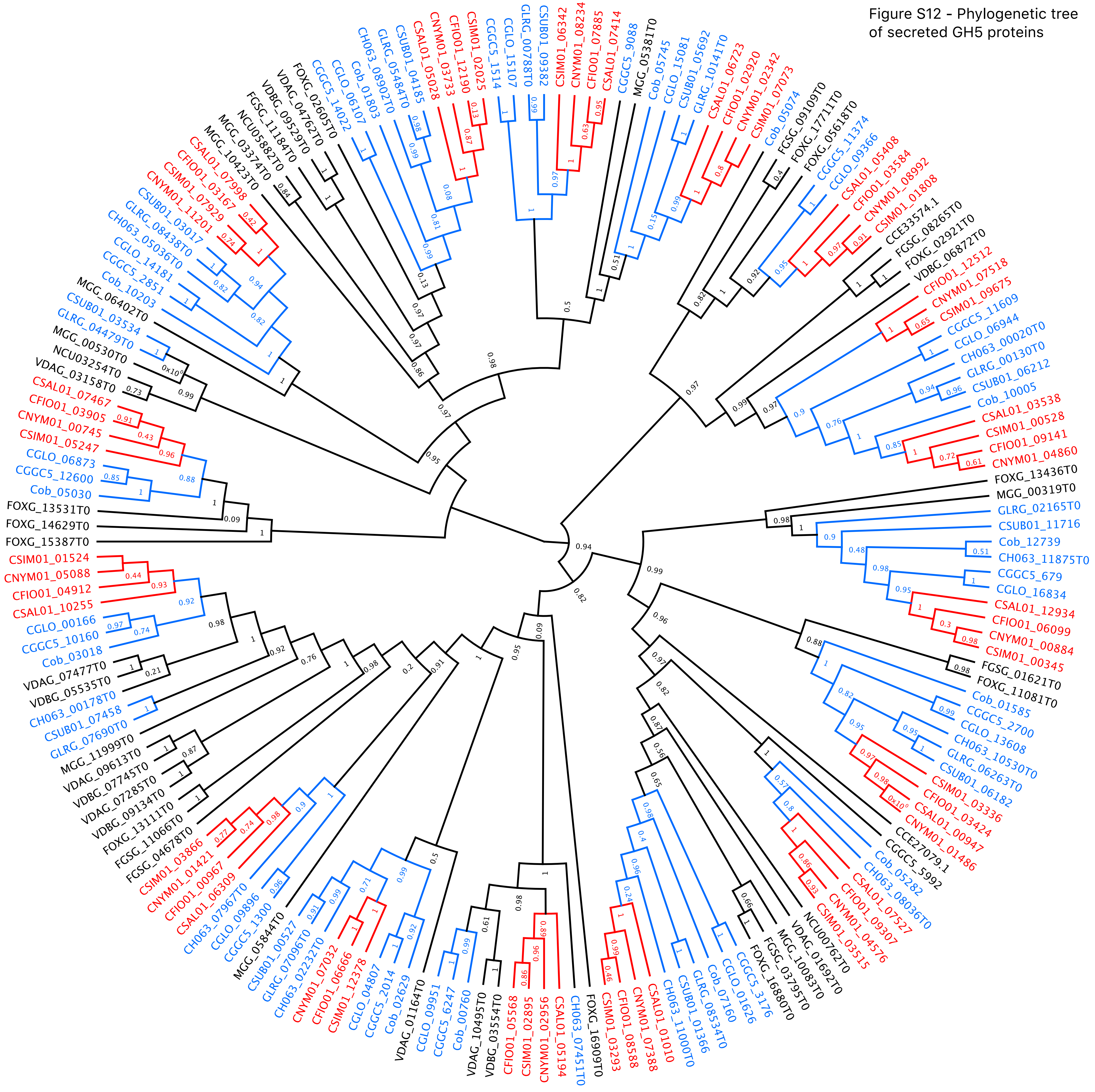

Figure S13 - Phylogenetic tree of secreted GH43 proteins

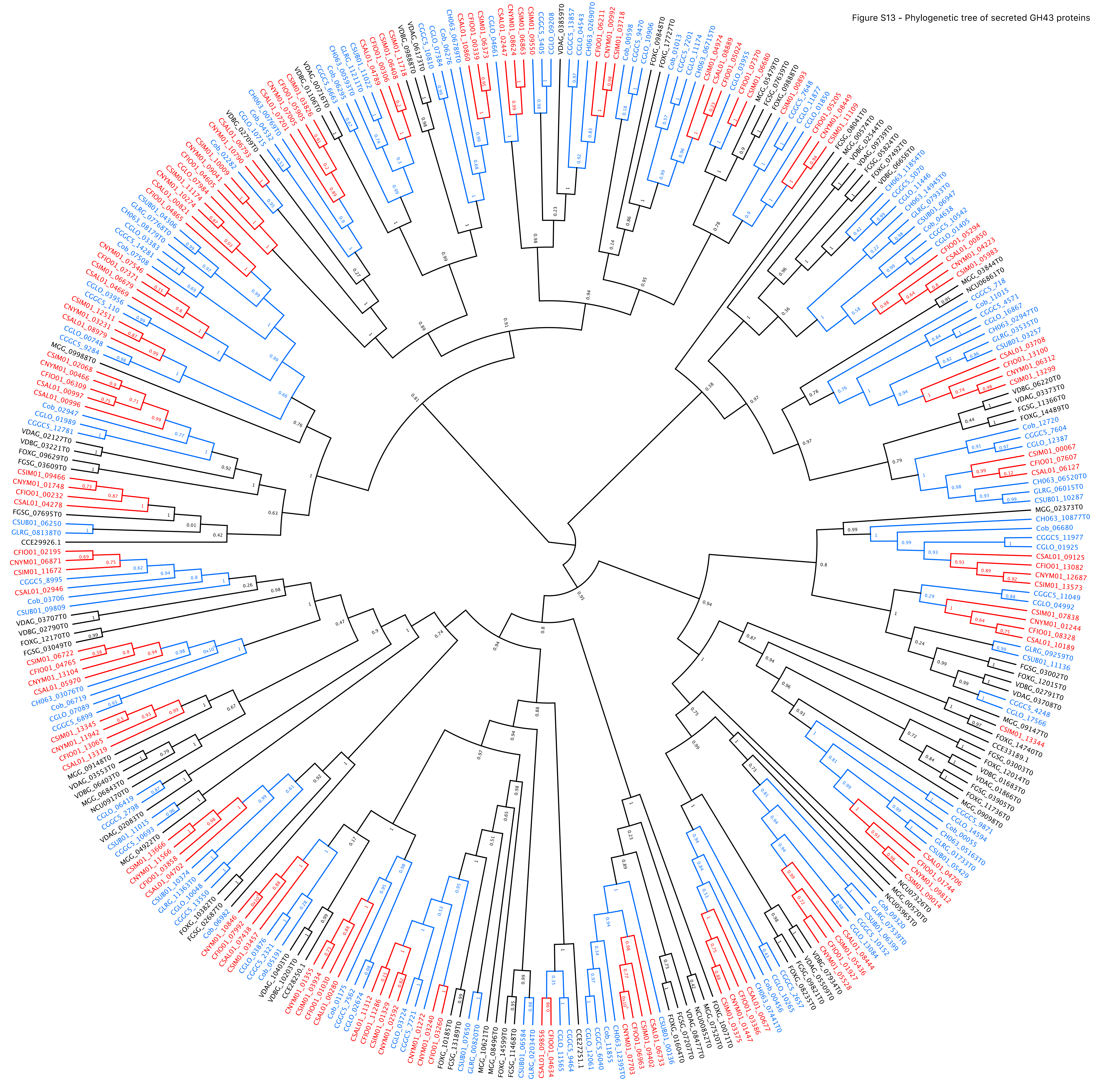

Supplement: Additional file 5: Figures S2–S13. — Figures S2 and S3. Clustering of secreted carbohydrate-active enzyme (S2) and peptidase (S3) families encoding genes identified in this study. Numbers of genes in each row were normalized using MeV 4.8.1. Hierarchical clustering of genes and species was performed and visualized using the package “pheatmap” 1.0.8 within R. Figures S4–S13. Phylogenetic trees of secreted proteins belonging to specific class of peptidases (Figure S4. A01A; Figure S5. S10; Figure S6. M43B; Figure S7. M35) and carbohydrate-active (Figure S8. AA3; Figure S9. AA7; Figure S10. CE10; Figure S11. CE16; Figure S12. GH5, Figure S13. GH43) enzyme families identified in the genomes analyzed in this study. Proteins were aligned with MAFFT and trees were inferred using the FastTree algorithm implemented in Geneious 8.1.4. Protein names in red are from CAsc species, Protein names in blue are from other Colletotrichum spp. (PDF 8509 kb) [file 12864_2016_2917_MOESM5_ESM.pdf]
